# Transient Interdomain Interactions Modulate the Monomeric Structural Ensemble and Self‐Assembly of Huntingtin Exon 1

**DOI:** 10.1002/advs.202501462

**Published:** 2025-04-28

**Authors:** Priyesh Mohanty, Tien Minh Phan, Jeetain Mittal

**Affiliations:** ^1^ Artie McFerrin Department of Chemical Engineering Texas A&M University College Station TX 77843 USA; ^2^ Department of Chemistry Texas A&M University College Station TX 77843 USA; ^3^ Interdisciplinary Graduate Program in Genetics and Genomics Texas A&M University College Station TX 77843 USA

**Keywords:** huntingtin, molecular dynamics simulation, polyglutamine

## Abstract

Polyglutamine (polyQ) tract length expansion (≥ 36 residues) within the N‐terminal exon‐1 of Huntingtin (Httex1) leads to Huntington's disease, a neurodegenerative condition marked by the presence of intranuclear Htt inclusions. Notably, the polyQ tract in Httex1 is flanked by an N‐terminal coiled‐coil domain –N17 (17 amino acids), which promotes the formation of soluble oligomers and brings the aggregation‐prone polyQ tracts in close proximity. However, the molecular mechanisms underlying the conversion of soluble oligomers into insoluble β‐rich aggregates with increasing polyQ length, remain unclear. In this study, extensive atomistic molecular dynamics (MD) simulations (aggregate time ≈0.7 milliseconds) are performed to uncover the interplay between structural transformation and domain “cross‐talk” on the conformational ensemble and oligomerization of Httex1 due to polyQ expansion. Notably, MD‐derived ensembles of N17‐Q_n_‐P_5_ monomers validated against NMR indicated that in addition to elevated α‐helicity, polyQ expansion also favored transient, interdomain (N17/polyQ) interactions which resulted in the emergence of β‐sheet conformations. Further, interdomain interactions modulated the stability of N17‐mediated polyQ dimers and promoted a heterogeneous dimerization landscape. Finally, it is observed that the intact C‐terminal proline‐rich domain (PRD) promoted condensation of Httex1 through self‐interactions involving its P_10_/P_11_ tracts while also interacting with N17 to suppress its α‐helicity.

## Graphical abstract

Polyglutamine (polyQ) tract expansion (≥ 36 amino acids) within the N‐terminal region of the Huntingtin protein (Httex1) causes Huntington's disease (HD), for which the underlying causes are not well‐understood. The authors performed computer simulations to understand the cause of HD at the molecular level. They observed that polyQ length expansion favors interdomain interactions which can lead to disease‐related β‐structures of Httex1 and destabilize its soluble oligomeric forms.

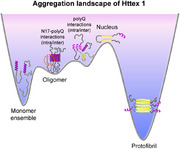

## 1 Introduction

There are nine polyglutamine (polyQ)‐associated neurodegenerative disorders that are known to arise as a result of CAG trinucleotide repeat expansion within the polyQ‐encoding tract of a specific gene.^[^ ^1^, ^2^, ^3^ ^]^ Huntington's disease (HD) is a polyQ‐related disorder marked by the presence of intranuclear inclusions (aggregates) of the protein – Huntingtin (Htt) in striatal neurons.^[^ ^4^, ^5^ ^]^ At present, there is no cure available for HD.^[^ ^6^, ^7^ ^]^ Huntingtin (Htt) is a 348 kDa multifunctional protein that shuttles between the nucleus and cytoplasm and is broadly involved in the development of the nervous system, brain‐derived neurotrophic factor (BDNF) production and transport, and cell adhesion.^[^ ^8^ ^]^ The loss of native Htt function and its toxic gain‐of‐function via length expansion and aggregation of the polyQ tract (>36 residues) within the N‐terminal Htt exon‐1 (Httex1) is associated with HD.^[^ ^9^, ^10^ ^]^ The length of polyQ expansion inversely correlates with the age of disease onset.^[^ ^11^ ^]^ Notably, Httex1 fragments generated through proteolytic cleavage are observed in intranuclear inclusions^[^ ^12^ ^]^ and in vivo expression of mutant Httex1 was found to reproduce the key features of HD in mice models.^[^ ^13^, ^14^ ^]^ Not surprisingly, the structural and conformational changes associated with polyQ expansion^[^ ^15^, ^16^, ^17^ ^]^ and the precise role of flanking regions in Httex1 oligomerization,^[^ ^18^ ^]^ fibrillar aggregation^[^ ^19^ ^]^ and cellular toxicity^[^ ^20^ ^]^ have been the subject of intense debate and investigations.^[^ ^21^, ^22^ ^]^

**Figure 1:**
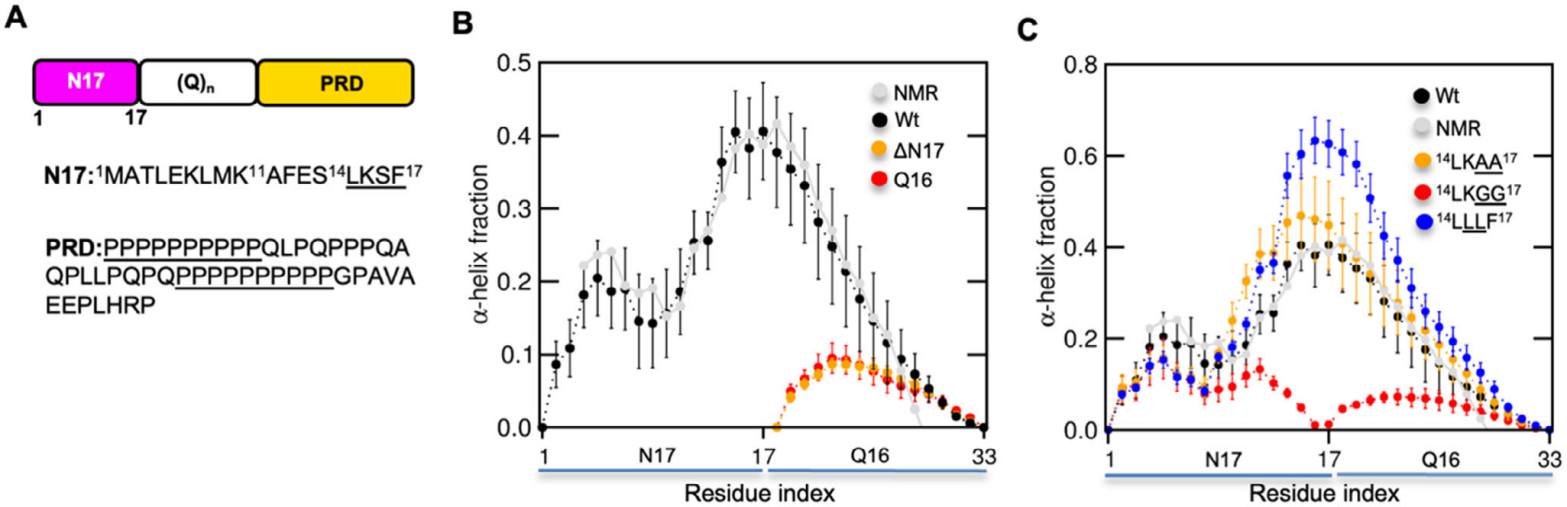
Validation of N17‐Q_16_‐P_5_ ensemble against solution NMR data and assessing the effect of N17 (^14^LKSF^[^ ^17^ ^]^) mutants on polyQ helicity. A) Schematic showing the organization of Httex1 into three regions – N17, polyQ, and PRD (top), and the corresponding sequences of N17/PRD domains (bottom). The length of polyQ region in the wild‐type Huntingtin protein is ≈23 residues. B) Comparison of per‐residue α‐helix fractions for N17‐Q_16_‐P_5_ wild‐type and its deletion constructs with respect to NMR. Helical fractions were computed using the DSSP algorithm and compared to SSP scores for the helical propensity (grey) computed from experimental ^13^C chemical shifts. Error bars denote std. An error of the mean was calculated over three independent trajectories. C) Same as in panel B for wild‐type and ^14^LKSF^[^ ^17^ ^]^ mutants.

Httex1 is an intrinsically disordered polypeptide (91 amino acids) comprising of i) an N‐terminal α‐helical domain (N17: 17 amino acids), a central polyQ tract which is aggregation‐prone and a C‐terminal proline rich domain (PRD: 51 amino acids) (**Figure** **1**A). The polyQ‐flanking domains (N17/PRD) exert opposing effects on fibrillar aggregation; N17 promotes fibrillation and suppresses the formation of non‐fibrillar species, while PRD generally disfavors aggregation and promotes the formation of soluble oligomers.^[^ ^19^, ^23^, ^24^, ^25^ ^]^ In terms of the classical nucleation model for polyQ amyloid formation,^[^ ^26^ ^]^ Wetzel and colleagues determined that the size of the critical nucleus required to template the formation of β‐rich aggregates of dilysine‐flanked polyQ peptides in vitro depends upon the length of polyQ: the nuclei for >Q_25_ are monomeric while smaller fragments are observed to form larger nuclei (n = 2–4).^[^ ^27^ ^]^ These findings were further corroborated in a computational study by Wolynes and co‐workers based on simulated free energy landscapes of polyQ aggregation computed using a coarse‐grained protein model.^[^ ^28^ ^]^ The study indicates that shorter polyQ monomers (e.g., Q_20_) prefer an extended conformation and a trimeric nucleus (n^*^≈3), while longer monomers (e.g., Q_30_) can preferably adopt a β‐hairpin conformation and undergo aggregation through a monomeric nucleus (n^*^≈3). Computational modeling also suggests that the nucleation of smaller polyQ fragments promotes elongation and fibrillation by reducing the conformational entropy of interacting monomers in a concentration‐dependent manner.^[^ ^29^ ^]^ Recently, Halfmann and colleagues confirmed the formation of a monomeric nucleus in vivo for the pathogenic polyQ_60_ fragment and determined its structural features using an integrative modeling approach.^[^ ^30^ ^]^

N17 significantly enhances the rate of polyQ fibrillation in vitro and in vivo through a complex, multistep mechanism involving small (low molecular weight) prenucleation oligomers which undergo further rearrangement to promote amyloid nucleation.^[^ ^31^, ^32^ ^]^ A kinetically competing, N17‐independent aggregation pathway also exists for Huntingtin exon 1 which resembles that of simple polyQ and dominates upon disruption of N17 association or its partial excision.^[^ ^33^ ^]^ Notably, solid‐state NMR experiments concluded that the core of Httex1 fibrils was structurally similar to simple polyQ fragments, comprising of an inter‐digitating hydrogen bond network formed between adjacent layers of polyQ β‐hairpins through glutamine sidechains while the flanking N17 remains helical.^[^ ^34^, ^35^, ^36^ ^]^ While higher‐order oligomerization through N17 significantly increases the local concentration of polyQ tracts (“proximity” model), such a mechanism alone fails to explain the enhanced rate of polyQ aggregation as forcing a high local concentration of polyQ by fusing it to homo‐oligomeric partners suppresses its fibrillar aggregation in vitro^[^ ^37^ ^]^ and in vivo.^[^ ^30^ ^]^ Two additional models were proposed to explain the conversion of Httex1 oligomers into aggregates via polyQ expansion^[^ ^37^ ^]^: i) “transformation” model, i.e., the propagation of N17 helical secondary structure into the polyQ tract, and, ii) the “domain cross‐talk” model, i.e., tertiary interdomain interactions between N17 and polyQ domains. A model of Httex1 amyloid aggregation based on photocross‐linking experiments proposes that long‐range N17/polyQ interactions result in a conformational switch which promotes rearrangement of the initial N17‐mediated oligomers to form aggregation‐competent nuclei.^[^ ^32^ ^]^

The relevance of interdomain interactions for Httex1 aggregation is further supported by in vivo experiments where the selective binding of a polyQ β‐hairpin antibody to mutant Httex1 oligomers^[^ ^38^ ^]^ was favorable when at least one of the flanking regions (N17 or PRD) was present.^[^ ^23^ ^]^

Nevertheless,^[^ ^42^ ^]^ the substantially faster aggregation of longer Httex1 constructs (>Q_7_) renders them less amenable to high‐resolution NMR experiments for studying conformational exchange and detecting interdomain interactions occurring with oligomeric assemblies.^[^ ^39^, ^40^ ^]^ Alternatively, molecular dynamics (MD) simulations have the potential to complement solution experiments and provide a high‐resolution picture of the conformational landscape and underlying mechanisms that facilitate the conversion of α‐helical oligomers into amyloid aggregates. Toward this goal, numerous studies have attempted to investigate the structural and conformational changes in Httex1 monomers upon polyQ expansion using atomistic MD simulations.^[^ ^41^ ^]^ Nevertheless, these studies report conflicting results regarding the nature of pathogenic conformations due to: i) inherent imbalances in secondary structure propensities (i.e., relative stabilities of α‐helical versus β‐conformations) for commonly‐used protein force fields^[^ ^42^, ^43^, ^44^ ^]^ and, ii) the tendency to generate excessively collapsed ensembles for unfolded and disordered proteins when simulated in implicit or primitive (three‐site) explicit water models.^[^ ^45^, ^46^, ^50^, ^51^, ^52^, ^53^ ^]^

In this study, we performed extensive MD simulations (aggregate time ≈0.7 milliseconds) to evaluate the role of interdomain interactions on the monomeric structural ensemble, oligomerization, and condensation of Httex1. Using a state‐of‐the‐art protein force field,^[^ ^47^ ^]^ our simulation‐derived ensembles of N17‐polyQ constructs with a minimal five‐residue C‐terminal polyproline tract (P_5_) showed good agreement with residue‐specific helical populations inferred from recent NMR experiments which employed site‐specific isotope labeling of polyQ tract residues.^[^ ^48^, ^49^ ^]^ With increasing polyQ length, we observed longer α‐helices extending further into the polyQ tract along with the emergence of transient β‐sheet conformations (<2% total population). Importantly, stable β‐sheet conformations were not observed for a Q_46_ polypeptide on a comparable simulation timescale, despite its low propensity for α‐helix formation. These findings imply that interactions between N17 and polyQ domains are directly implicated in enhancing the formation of β‐sheet conformations which in turn could serve as precursors in the folding pathway enroute to the nucleus. Next, we investigated the interplay between helical transformation (i.e., elevated polyQ helicity) and interdomain interactions on the dimerization landscape of Httex1. We observed that an increase in polyQ length (7 to 16) allowed transient, intra, and intermolecular N17/polyQ interactions to effectively outcompete the stabilizing effect of α‐helical transformation and promote a heterogenous dimer ensemble. Finally, we observed that while the presence of an intact C‐terminal PRD does not alter the intrinsic α‐helical propensity of the N17‐Q_16_‐PRD monomer (cis), it promotes condensation^[^ ^50^, ^51^, ^52^ ^]^ through intermolecular interactions involving P_10_/P_11_ tracts while also interacting with N17 (trans) to suppress its α‐helicity. In conclusion, our results support a model wherein inter‐domain interactions can exert opposing effects on Httex1 aggregation by both promoting the formation of aggregation‐competent nuclei^[^ ^32^ ^]^ and condensation to limit aberrant phase transitions.^[^ ^53^, ^54^ ^]^

## 2 Results

### 2.1 Structural Validation of N17‐Q_16_‐P_5_ Simulation Ensemble Against Solution NMR

The α‐helical structure of N17 is associated with enhanced oligomerization and aggregation of Httex1 compared to their simple polyQ counterparts. Recent structural studies employing NMR spectroscopy with site‐specific isotope labeling resolved the residue‐level α‐helical propensities of amino acids within the N17 and polyQ regions in Httex1 constructs with increasing polyQ length below (Q_16_) and above the pathogenic threshold (Q_46_).^[^ ^48^, ^55^ ^]^ Such high‐resolution, residue‐specific information serves as an invaluable reference data set for the structural validation of Httex1 ensembles generated by MD force fields. We chose a balanced protein force field–AMBER03ws,^[^ ^47^ ^]^ to generate conformational ensembles of N17‐Q_n_‐P_5_ monomers and dimers using multi‐microsecond and enhanced sampling simulations (Methods, Table S1, Supporting Information). Previous studies by us and others have firmly established the ability of AMBER03ws to accurately describe the global dimensions and local structural features of a wide variety of intrinsically disordered polypeptides (IDPs).^[^ ^56^, ^57^ ^]^ We first performed multi‐microsecond simulations of the N17‐Q_16_ which includes a minimal C‐terminal polyproline tract consisting of five residues (P_5_). It is worth noting that several of the key experimental studies aimed at deciphering the aggregation pathway of Httex1 have exclusively employed C‐terminal (PRD) truncated constructs.^[^ ^33^, ^58^ ^]^ It was subsequently shown that the oligomerization and aggregation pathway of Httex1 and the C‐terminal truncation variant are essentially identical under in vitro conditions.^[^ ^59^ ^]^ Further, site‐specific isotope labeling NMR experiments indicate that disruption of the structural connectivity between polyQ and the adjacent PRD has only a modest impact on the helicity (slight increase) of the upstream polyQ sequence.^[^ ^48^ ^]^

The mean probability distribution for the radius of gyration across three independent trajectories exhibited low standard errors, implying convergence of the N17‐Q_16_‐P_5_ ensembles in terms of their global dimensions (Figure S1A, Supporting Information). To validate the structural accuracy of the N17‐Q_16_‐P_5_ ensemble, we first compared it with per‐residue α‐helical propensities computed from ^13^C chemical shifts using the SSP algorithm.^[^ ^60^ ^]^ Overall, mean per‐residue helical fractions of N17‐Q_16_‐P_5_ were found to be in excellent agreement with NMR across both N17 and Q_16_ regions (Figure 1B). Further, the direct comparison of ^13^C_α/β_ chemical shifts predicted using the SPARTA+ program (See Experimental Section) indicated that the root mean square deviation (RMSD) over all residues with respect to experiment is within the prediction error (Figure S1B, Supporting Information). The comparison of per‐residue ^13^C secondary chemical shift difference (SCSD) with NMR shows excellent quantitative agreement (≤0.5 ppm) for several residues in the N17 region (Figure S1C, Supporting Information). Further, we observed a gradual decay in polyQ SCSD from the start (Q18) to the end position (Q33), a trend which is qualitatively consistent with the experiment. As negative controls, we also simulated Q_16_‐P_5_ and Q_16_ fragments; both exhibited substantially lower α‐helicity compared to N17‐Q_16_‐P_5_ which is consistent with the experiment (Figure 1B).

NMR experiments indicated that a four residue motif in N17 (^14^LSKF^[^ ^17^ ^]^) structurally connects N17 and the downstream polyQ residues.^[^ ^48^ ^]^ Notably, these experiments revealed that ^14^LKSF^[^ ^17^ ^]^ uniquely favored the formation of bifurcated hydrogen bonds involving glutamine sidechains (i→i‐4) in the adjacent polyQ region (aa:18‐24) which could contribute to the elevated α‐helical propensity.^[^ ^48^ ^]^ Similarly, an L_4_ motif flanking the Androgen receptor polyQ tract was found to promote bifurcated hydrogen bonds which stabilized its α‐helical structure.^[^ ^61^ ^]^ Based on site‐specific labeling of a few glutamine residues, it was observed that substitution of the wild‐type motif – ^14^LKSF^[^ ^17^ ^]^ to either ^14^LKAA^[^ ^17^ ^]^ or ^14^LLLF^[^ ^17^ ^]^ enhanced helical stability while ^14^LKGG^[^ ^17^ ^]^ substitution significantly reduced the helicity of the polyQ region. We simulated all three ^14^LKSF^[^ ^17^ ^]^ variants to generate the per‐residue helicity profiles of all mutants and assessed whether our force field could correctly capture the expected trends in their α‐helicity profiles. The helix‐promoting mutants showed an increase in α‐helicity (Figure 1C) starting from the C‐terminal region of N17 (aa:11‐17) and leading into the polyQ region (aa:18‐23). In contrast, ^14^LKGG^[^ ^17^ ^]^ disrupted the structural connectivity between N17 and polyQ regions, resulting in a complete loss of α‐helical structure in the Q_16_ tract (Figure 1C) and a corresponding increase in the population of coil conformations (Figure S1D, Supporting Information). We also analyzed our ensembles for the prevalence of bifurcated hydrogen bonds in the polyQ tract among α‐helical conformers with helix fraction greater than 30%. While the overall occupancy of these hydrogen bonds was low, the observed trend positively correlated with their residue‐level α‐helical fractions: ^14^LLLF^[^ ^17^ ^]^ variant showed higher hydrogen bond occupancies (>5%) in the central region (Q18/19) compared to ^14^LKAA^[^ ^17^ ^]^ and the wild‐type sequence–^14^LKSF^[^ ^17^ ^]^ (Figure S1E, Supporting Information).

Overall, the above comparison of N17‐Q_16_ ensembles with high‐resolution solution NMR data firmly establishes the suitability of our chosen model to generate an accurate structural ensemble of N17‐Q_16_‐P_5_ and predict the effect of ^14^LKSF^[^ ^17^ ^]^ point mutations on α‐helix stability. These observations collectively encouraged us to analyze the mechanisms of helix initiation and propagation, as described in the following section.

### 2.2 Mechanism of α‐Helix Nucleation and Propagation into the polyQ Tract

**Figure 2:**
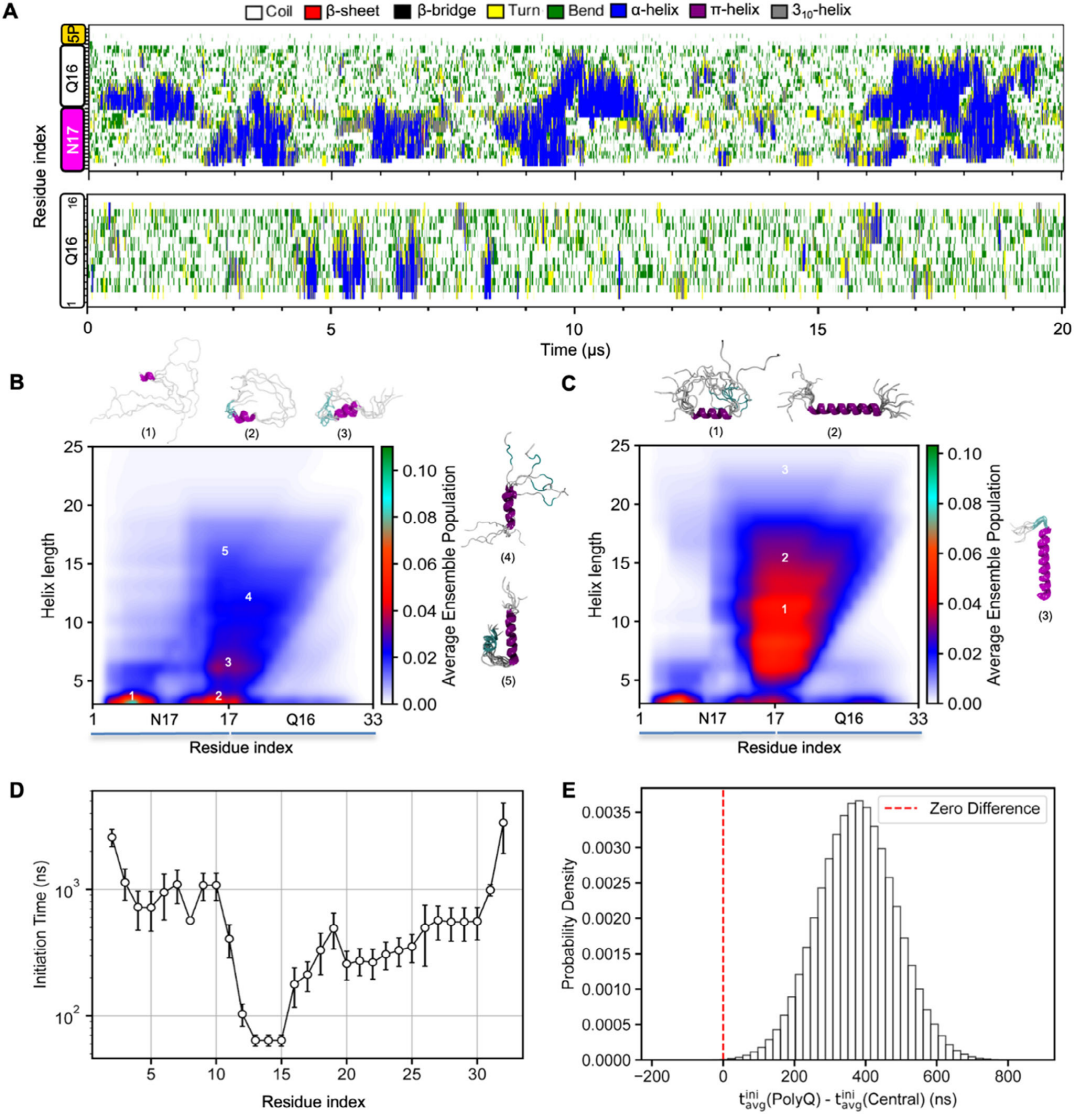
Structural cooperativity between N17 and polyQ domains. A) Secondary structure variation as a function of time in a representative N17‐Q_16_‐P_5_ (top) and Q_16_ (bottom) trajectory. B) SS‐map of N17‐Q_16_‐P_5_ wild‐type computed from an aggregate trajectory (≈63 µs) indicating the probability of various helical lengths across N17 and polyQ regions. Representative α‐helical conformations corresponding to numbered regions of the SS‐map are shown in cartoon representation. C) Same as in E for ^14^LLLF^[^ ^17^ ^]^ mutant. D) Helix initiation times for each residue along the peptide sequence. Initiation time represents the earliest simulation frame (in ns) at which each residue adopts a helical conformation. Error bars indicate standard errors of the mean computed across three independent simulation trajectories. Residues in the central region (approximately residues 11–17) exhibit notably lower initiation times, indicating earlier helix formation compared to residues in the polyQ tract region (higher residue numbers). E) Histogram depicting the Bayesian‐inspired Monte Carlo sampling of initiation time differences between the polyQ region and the central region (Δt = t_avg_(polyQ) – t_avg_(central)). The red dashed line at zero indicates no difference between regions. Positive values demonstrate that the central region consistently forms helices earlier than the polyQ region.

The high structural accuracy of our N17‐Q_16_‐P_5_ ensemble motivated us to analyze the mechanisms of helix nucleation and propagation into the Q_16_ region which accounts for the observed residue‐level α‐helical populations. Accordingly, we first visualized the variation in secondary structure across both domains over the time course of the three independent trajectories (**Figure** **2**A; Figure S2A, Supporting Information). Upon careful inspection, we observed that α‐helices generally appeared to nucleate in the region spanning the C‐terminal N17 region (aa:11–17) and the adjacent Q_16_ tract (aa:18–23) and could further propagate in a bidirectional manner to form longer helices which persisted for several microseconds. However, these helices were less stable and did not persist for more than a microsecond. The N‐terminal region of N17 (aa:3–10) formed transient helical structures (<1 µs) which were weakly associated with the formation of longer helices, suggestive of its structural decoupling from the downstream N17‐polyQ sequence. Occasionally, N‐terminal N17 also appeared to act as a nucleation site while also forming short α‐helices which partially extended into the C‐terminal N17 region (Figure S2A, Supporting Information, bottom panel). In both N17‐Q_16_‐P_5_ and Q_16_ trajectories, short α‐helices (<8 residues) appeared within different stretches of polyQ sequence and usually persisted for less than 0.2 µs. In the case of Q_16_ trajectories, longer α‐helices were also observed, although these typically did not persist beyond 1 µs, highlighting the critical requirement of the N17 domain for stable α‐helix nucleation and propagation into the polyQ tract (Figure 2A; Figure S2B, Supporting Information).

The observations from the time course analysis of N17‐Q_16_‐P_5_ are summarized in the form of a α‐helical secondary structure (SS) map^[^ ^62^ ^]^ which describes the sequence‐level cooperativity underlying α‐helix formation by reporting on the helical populations for a range of possible lengths. The SS map reveals that α‐helical populations were the highest for single turns (<5 residues) in either N‐ or C‐terminal region of N17, followed by a short α‐helix in the central region (aa:11‐23) which can propagate into further into the polyQ tract to form longer helices (Figure 2B). In comparison, the ^14^LLLF^[^ ^17^ ^]^ variant showed a clear increase in the stability of longer α‐helices (upto 15 residues) initiated from the central region as indicated by their higher populations in the SS map (Figure 2C). The structural decoupling of the N‐terminal N17 region was evident in N17‐Q_16_‐P_5_ wild‐type based on its reduced prevalence in longer helices (>8 residues) compared to the downstream N17‐polyQ sequence and even more pronounced in the case of the ^14^LLLF^[^ ^17^ ^]^ variant. Overall, the time course analysis of secondary structure formation and SS maps suggests a mechanism of helix initiation which is favored from the central region (aa:11–23) and its propagation into the polyQ tract leads to longer helices. Further, the N‐terminal N17 region (aa:3–10) has a low α‐helix propensity and can be structurally uncoupled from the downstream sequence.

To determine the temporal sequence of α‐helix formation across the N17/polyQ domains, we calculated the helix initiation time per residue, we first determined the helix initiation time for each residue by analyzing the DSSP secondary structure data over the three independent simulation trajectories. The initiation time was defined as the earliest time (frame) at which each residue adopted an α‐helical conformation while residues never adopting a helical structure were marked accordingly. From this data, we observed a consistent trend wherein residues in the central region (end of the N17 region, aa:11–17) displayed significantly lower initiation times (<100 ns) compared to those within the polyQ tract (≈10^2^ ns or higher) (Figure 2D). This trend strongly suggests that the central‐region acts as the nucleation site for helix formation, with subsequent propagation into the polyQ tract. To quantitatively assess the reliability of this conclusion, we implemented a Bayesian‐inspired Monte Carlo sampling approach (See Methods). An analysis of the histogram of initiation time differences (Δt = t_avg_(polyQ)–t_avg_(central)) computed from our sampling approach consistently showed positive values, centered around ≈400 ns (Figure 2E), implying that the polyQ region consistently lagged behind the central region in helix initiation. This robust statistical analysis further provides strong quantitative support for our original conclusion that the central region acts as a nucleation site, with subsequent helix propagation into the polyQ tract.

Based on extensive evaluation and modification, Robustelli et al.^[^ ^57^ ^]^ proposed that the AMBER99SB‐disp forcefield may represent a suitable force field choice for modeling IDP ensembles. Hence, we also simulated the N17‐Q_16_‐P_5_ construct with AMBER99SB‐disp and assessed its ability to accurately reproduce the NMR‐derived helical fractions (Figure S2, Supporting Information). As evident from the per‐residue α‐helical fractions (Figure S3A, Supporting Information), the AMBER99SB‐disp ensemble exhibits two major issues compared to NMR: i) it incorrectly predicts the position for peak α‐helicity and, ii) overestimates the ɑ‐helical fraction (by ≈5–15%) across the entire polyQ tract. Further, visualization of the secondary structure variation over three independent trajectories (≈15 µs each) reveals several instances of stable α‐helix formation (>1 µs) across the polyQ tract which was independent of N17 α‐helicity (Figure S3B, Supporting Information) and not observed in case of the AMBER03ws ensemble. Finally, the ensemble SS‐map indicates weaker structural cooperativity between N17 and polyQ regions for α‐helix propagation across these regions (Figure S3B,C, Supporting Information), in stark contrast to AMBER03ws. In addition, the AMBER99SBws‐STQ which was developed by us for accurate simulations of prion‐like sequences^[^ ^63^ ^]^ (enriched in Ser, Thr, and Glu) considerably underestimated the ɑ‐helicity of the polyQ region.^[^ ^49^ ^]^ To remedy this issue, we recently reported that a slight readjustment of the torsional constant (k_Ψ_) for glutamine led to an increase in polyQ helicity and improved agreement with NMR SSP scores.^[^ ^64^ ^]^ Overall, we concluded that AMBER03ws provided the most accurate description of the N17‐Q_16_‐P_5_ structural ensemble and hence, was chosen to generate the simulation ensembles of all other polyQ constructs appearing in this study.

### 2.3 Emergence of β‐Sheet Conformations upon polyQ Length Expansion is Driven by Weak Inter‐Domain Interactions

In vitro experiments indicate that the critical nucleus size for polyQ aggregation reduces from a multimer (dimers to tetramers) to a monomer in the case of longer fragments (>Q_25_).^[^ ^27^ ^]^ Based on these observations, an outstanding question is how polyQ expansion influences the structural ensemble of the Httex1 monomer and induces conformational changes in the polyQ tract to favor aggregation. Current evidence suggests that the formation of β‐hairpin structure in expanded Httex1 strongly favors amyloid aggregation. First, a monoclonal antibody (3B5H10) recognizes a two‐stranded β‐hairpin conformation in the polyQ tract of mutant Httex1 monomer and its formation in vivo effectively predicts neurodegeneration.^[^ ^65^, ^66^ ^]^ Second, the aggregation rate of a non‐pathogenic Httex1 fragment can be enhanced to levels comparable to that of a pathogenic fragment through the introduction of short β‐hairpin‐forming motifs into polyQ sequences.^[^ ^67^ ^]^ Despite the importance of β‐hairpin conformations in Httex1 aggregation and toxicity, NMR and FRET experiments indicate an increase in α‐helicity and rigidification of the polyQ tract with length expansion.^[^ ^49^, ^55^, ^68^, ^69^ ^]^ Further, the elevated helicity in the pathogenic Httex1‐Q46 also correlated with an enhanced rate of aggregation in vitro and in vivo.^[^ ^49^ ^]^

**Figure 3:**
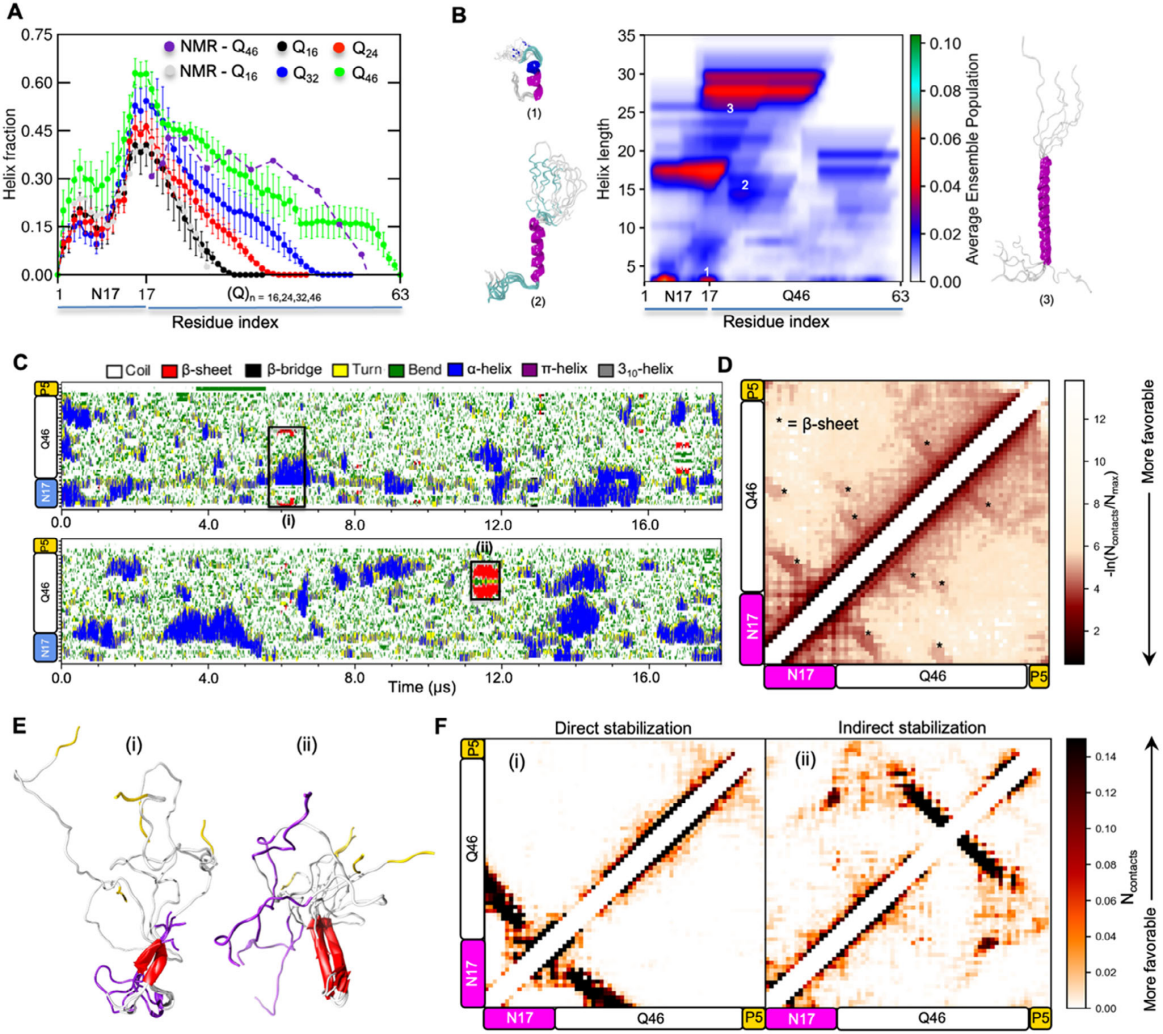
Effect of polyQ length expansion on N17‐Q_n_‐P_5_ structural ensemble. A) Comparison of per‐residue α‐helix fractions for N17‐polyQ_n_‐P_5_ constructs with increasing polyQ length (Q_16‐46_) against NMR‐derived helical propensities (SSP scores). Error bars denote std. The error of the mean was calculated over three independent trajectories for N17‐Q_16‐32_ constructs and over four intervals (150 ns each) of the PT‐WTE replica trajectory (293 K) for N17‐Q_46_‐P_5_. B) SS‐map of N17‐Q_46_‐P_5_ wild‐type (left) from the PT‐WTE aggregate trajectory (600 ns) indicating the probability of α‐helices formed of varying lengths across N17 and polyQ regions. Representative helical conformations corresponding to numbered regions of the SS‐map are shown in cartoon representation. C) Secondary structure variation as a function of time for two representative N17‐Q_46_‐P_5_ trajectories showing the formation of two‐stranded β‐sheet structures (black boxes). D) 2D intramolecular contact maps calculated over six N17‐Q_46_‐P_5_ independent trajectories indicating the low population of β‐conformations (marked as *) relative to α‐helices. E) Representative β‐sheet structures (5 in total) from the trajectory periods highlighted in C (black boxes) involving either direct (i) or indirect (ii) contacts between the N17 and Q_46_ domains. The coloring scheme for the structures is as follows: N17 – purple, Q_46_ – white P_5_ – Gold, β‐strand – red. F) Pairswise 2D contact maps calculated for the two β‐sheet structural ensembles shown in (E).

To address the underlying interplay between α‐helix and β‐structure formation in the context of polyQ expansion, we performed multi‐microsecond simulations (Table S1, Supporting Information) of N17‐polyQ fragments with increasing polyQ length: Q_24/32/46_ and compared their structural ensembles. Consistent with NMR experiments, the per‐residue α‐helix fractions increased in a polyQ length‐dependent manner (Q_16_ to Q_46_) (**Figure** **3**A). The elevated α‐helix fraction was predominantly associated with residues beyond the central polyQ tract, implying long‐range structural cooperativity across the N17 and expanded polyQ. In the case of N17‐Q_46_‐P_5_, however, multi‐microsecond simulations (aggregate duration ≈105 µs) yielded an ensemble with lower (15–20%) α‐helicity per‐residue in the polyQ tract compared to NMR estimates (Figure S4A, Supporting Information) despite the choice of diverse initial (coil and helical) conformations and convergence of R_g_ distributions across the two sets of trajectories (Figure S4B, Supporting Information). With parallel tempering simulations carried out in the well‐tempered ensemble (PT‐WTE, 750 ns per replica), we obtained a well‐converged ensemble (Figure S4C,D, Supporting Information) showing improved agreement with NMR for α‐helicity within the polyQ tract (Figure 2A). These observations highlight the difficulty associated with adequate sampling of structural transitions for IDPs with α‐helical propensity using multi‐microsecond simulations, due to long helix nucleation times.^[^ ^70^ ^]^ The computed SS map of the N17‐Q_46_‐P_5_ ensemble from the PT‐WTE temperature replica (293 K) clearly indicates the elevated populations of longer helices (25–30 residues) which formed via propagation from the central region as observed for N17‐Q_16_‐P_5_ (Figure 3B).

A closer inspection of the independent trajectories revealed the formation of transient two‐stranded β‐sheets (See Methods) for all three expanded N17‐polyQ constructs (Figure 3C; Figures S5,S6, Supporting Information) which was not observed in N17‐Q_16_‐P_5_ and visualized in the 2D intramolecular contact maps computed for each of the ensembles (Figure S7, Supporting Information, 3D). For N17‐Q_24_‐P_5,_ a two‐stranded β‐sheet structure formed in two trajectories (total population ≈0.5%) (Figure S5A, Supporting Information) while only a β‐bridge structure involving N17 and polyQ was also observed in another trajectory. For N17‐Q_32_, two out of three trajectories showed the formation of two‐stranded β‐sheet structures (total population ≈1.8%) (Figure S5B, Supporting Information). Among the six N17‐Q_46_‐P_5_ trajectories, β‐sheet conformations involving the polyQ tract were observed in five trajectories (six conformations) with an aggregate population of ≈1.9% (Figure 3D; Figure S6A,B, Supporting Information). Interestingly, half of the β‐conformations of N17‐Q_46_‐P_5_ were directly stabilized by hydrogen‐bonding with N17, and the other half involved hydrogen bonding within the polyQ region (Figure 3E‐F; Figure S5C, Supporting Information). Similar to the unbiased N17‐Q_46_‐P_5_ trajectories, a low population of β‐sheet conformations (1.1%) were also observed in the PT‐WTE 293 K replica trajectory (Figure S8, Supporting Information), further confirming their unstable nature compared to α‐helical and coil conformations. A summary of the β‐sheet conformer populations for the N17‐Q_n_‐P_5_ monomer ensembles described above is provided in Table S2 (Supporting Information).

The direct participation of N17 in the formation of β‐bridges/sheets (Figure 3F, left) raises a possibility that the emergence of β‐sheet conformations in N17‐Q_46_‐P_5_ may be enhanced through transient interdomain interactions. In support of this idea, the contact map of a β‐sheet conformation stabilized within the polyQ region also showed weak N17/polyQ interdomain interactions (Figure 3F, right). As stated earlier, the conformation‐specific Httex1 antibody (3B5H10) selectively binds to a two‐stranded β‐hairpin formed within the polyQ tract of pathogenic Httex1.^[^ ^66^ ^]^ Notably, the antibody bound to the polyQ tract in vivo only when either of the Httex1 flanking domains (N17/PRD) were present.^[^ ^23^ ^]^ These observations suggest that polyQ flanking regions such as N17 can modulate the polyQ conformational ensemble and promote the emergence of pathogenic conformations such as intramolecular β‐hairpins which favor amyloid aggregation.^[^ ^67^ ^]^ To directly test the idea that N17 promotes β‐conformations in the polyQ tract through interdomain interactions, we performed multi‐microsecond (triplicate) simulations of a simple Q_46_ polypeptide and compared it to the N17‐Q_46_‐P_5_ ensemble. The analysis of secondary structure variation over the time course of the Q_46_ trajectories (Figure S9, Supporting Information) indicated a near absence of β‐sheet conformations (Table S2, Supporting Information). These observations confirm the ability of interdomain N17/polyQ interactions to promote transient chain compaction and form potentially “toxic” β‐conformations.

### 2.4 The Interplay Between Inter‐Domain Interactions and Increased polyQ Tract α‐Helicity Modulates the Oligomerization Landscape of Httex1

**Figure 4:**
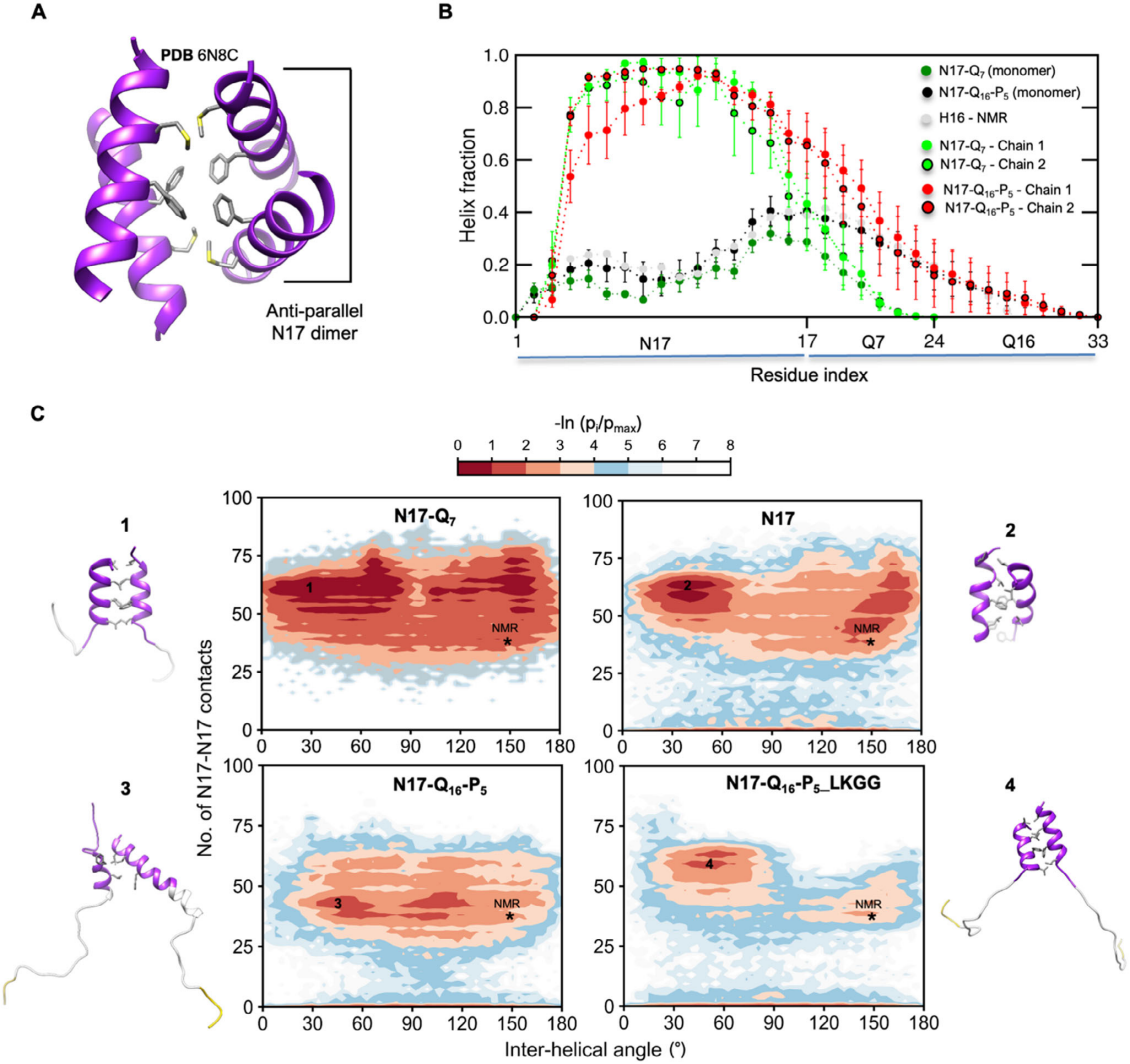
Competition between structural transformation and interdomain interactions shape the dimerization landscape of N17‐polyQ constructs. A) NMR structure of N17‐Q_7_ (H7) tetramer showing interactions (sidechains shown as sticks) in the hydrophobic core. The anti‐parallel dimer was extracted from the structure for MD simulations. B) Comparison of per‐residue α‐helix fractions for N17‐Q_7_/N17‐Q_16_‐P_5_ chains from dimer simulations against monomer simulations and NMR. Error bars denote std. The error of mean was calculated over six independent trajectories. The comparison indicates significantly elevated helicity of the N17 region in N17‐Q_7/16_ dimers compared to their respective monomers. C) 2D PMF (potential of mean force) plots as a function of two order parameters – (i) total number of intermolecular N17 contacts and (ii) the inter‐helical angle (°) between N17 domains, which characterize the association free energy landscape for four N17 dimer variants. The plots show the combined data derived from 6 independent dimer trajectories (≈2.3 µs) for each variant. The position of the NMR – N17 dimer (initial structure) is marked on the PMF plots as (*). Representative dimer MD structures from the basins marked (1, 2) in PMF plots are shown on the left with interfacial hydrophobic residues shown as grey sticks.

NMR studies indicate that the tetramerization of Httex1 via the helical N17 region is essential for efficient nucleation and fibrillation.^[^ ^39^ ^]^ Based on PRE measurements and simulated annealing,^[^ ^39^ ^]^ a structural model of α‐helical N17 tetramer (<1% population) was determined for a N17‐Q_7_ construct (high solubility) and found to comprise two anti‐parallel N17 dimer units stabilized by hydrophobic interactions (**Figure** **4**A). The formation of the tetrameric intermediate proceeds through weak dimerization (K_d_ ≈0.1–10 mM) as detected by NMR and analytical ultracentrifugation.^[^ ^39^ ^]^ The dimeric ensemble is proposed to comprise “productive” (i.e., anti‐parallel) dimers that can effectively assemble into nucleation‐competent tetramers and “non‐productive” dimers that block tetramerization. Interestingly, the conversion of prenucleation tetramers into amyloid fibrils remains unfavorable for polyQ tract length < 8 residues.^[^ ^58^ ^]^ Therefore, a mere increase in the local concentration of polyQ tracts through oligomerization is insufficient to explain the tetramer‐dependent aggregation pathway. Two additional phenomena were invoked based on in vitro experiments to explain the underlying mechanism^[^ ^37^ ^]^: i) structural transformation, i.e., an increase in α‐helical structure, and ii) domain “cross‐talk”, i.e., the emergence of weak inter‐domain interactions. However, the precise effects of these two phenomena on the oligomerization landscape of Htttex1 remain challenging to address using experimental techniques such as NMR due to the low population of oligomeric species and poor solubility of Httex1 disease mutants.

In order to tease apart the effects of structural transformation and inter‐domain interactions on the oligomerization landscape of Httex1, we first simulated two “productive” dimer variants extracted from the tetramer model as model systems: N17‐Q_7_ and N17‐Q_16_‐P_5_. To prepare the initial structures for dimer simulations, we modeled the polyQ sequences (Q_7/16_) into each monomer unit of the N17 dimer extracted from the tetrameric complex (Figure 4A). N17‐Q_7/16_ dimer ensembles showed an oligomerization‐dependent stabilization of α‐helical structure in N17 (Figure 4B; Figure S10A–C, Supporting Information) which is consistent with the CD and NMR experiments.^[^ ^39^, ^58^ ^]^ Notably, the expansion of the polyQ tract from 7 to 16 residues also promoted α‐helix propagation into the first few glutamine residues, as indicated by their elevated α‐helical populations (Figure 4B). The N17‐Q_7_ dimer maintained its stability over a microsecond timescale; none of the trajectories showed dissociation of the dimer (Figure 4C; Figure S11A, Supporting Information–top row, left) on the simulated timescale (≈2.3 µs per trajectory). To characterize the free energy landscape of the dimer variants, the 2D potential of mean force (PMF) plots were computed based on two order parameters: i) The total number of contacts between N17 domains of the monomers which distinguish between stable and weakly‐associated states, and ii) the inter‐helical (N17) angle to characterize the diversity of monomer orientation (Figure 4C). Interestingly, the bound dimer ensemble showed considerable heterogeneity, i.e., in addition to the antiparallel “native” orientation, additional energy basins (favorable regions) were observed for parallel orientations. We speculate that these alternate orientations may be representative of the “unproductive” dimers which are incompatible with higher‐order tetramerization.

PolyQ expansion from 7 to 16 appeared to reduce the “native” dimer stability as evident by the loss of numerous native‐like and parallel dimer energy basins (Figure 4C‐bottom row, left). Three out of six trajectories showed complete dissociation of the dimer followed by multiple weak reassociation events (Figure S11C, Supporting Information). The secondary dimers showed a reduced number of contacts (<25) between N17 domains and α‐helix stability (Figure S10B,C, Supporting Information) compared to the bound ensemble. To assess whether the domain “cross‐talk” model^[^ ^37^ ^]^ could explain the reduced stability of N17‐polyQ_7/16_, we computed contact maps of both N17‐polyQ_7_/Q_16_ dimer ensembles. In support of the domain “cross‐talk” model, contact map analysis indicated an extensive network of inter‐domain N17/polyQ interactions for N17‐polyQ_16_‐P_5_ (Figure S11E,F, Supporting Information).

To determine the effect of increased polyQ helicity on the stability of the bound dimer ensemble, we simulated two dimer variants: N17 and N17‐Q_16_‐P_5_ dimer variants with ^14^LKGG^[^ ^17^ ^]^ substitution.^[^ ^48^ ^]^ The N17 dimer was found to be highly unstable compared to N17‐Q_7_; five out of the six trajectories resulted in complete dissociation (Figure 4C, 311B–top row/right) due to the reduced α‐helicity of N17 in the absence of a downstream polyQ tract. Similarly, among the ^14^LKGG^[^ ^17^ ^]^ trajectories, five out of six trajectories showed complete dissociation followed by weak reassociation (Figure S11D, Supporting Information). Accordingly, the resulting dimerization landscape shows a lower population of stable N17‐mediated dimers (Figure 4C bottom row, right) and a concomitant increase in the population of weakly‐associated dimers. These results support the interpretation that structural transformation (i.e., increase in α‐helicity) of the monomer promotes N17 dimer stability and provides a plausible explanation for the reduced aggregation propensity of Httex1‐Q_46_^14^LKGG^[^ ^17^ ^]^ variant as observed in experiments.

The above findings enable us to conclude that an increase in polyQ length can promote transient intra‐ and intermolecular N17/polyQ interactions which counteracts the stabilizing effect of ɑ‐helical propagation into the polyQ tract, thereby promoting a heterogenous dimerization landscape. As a logical extension of this idea, we speculate that for efficient nucleation to occur within the tetrameric intermediate,^[^ ^39^ ^]^ polyQ‐mediated interdomain interactions (which are enhanced upon length expansion) must ultimately outcompete the stabilizing effect of increased polyQ α‐helicity on N17‐mediated oligomerization.

### 2.5 Proline‐Rich Domain (PRD) Modulates Httex1 Structure and Intermolecular Interactions within Liquid‐Like Assemblies

While the aggregation pathway of Httex1 and a C‐terminal construct lacking an intact PRD (P_10_ only) remains identical under in vitro conditions,^[^ ^59^ ^]^ PRD exerts a negative influence on the aggregation kinetics of Httex1 by increasing the relative stability of soluble oligomers compared to amyloid aggregates.^[^ ^23^ ^]^ Further, it was recently observed that the presence of PRD strongly promoted the liquid‐liquid phase separation^[^ ^53^ ^]^ of Httex1‐Q_25‐97_ under both in vitro and in vivo conditions when the latter is attached to a fluorescent protein tag.^[^ ^71^ ^]^ Interestingly, PRD was also found to suppress the emergence of amyloid aggregates from liquid‐like Httex1 condensates (i.e., a “liquid‐to‐solid” transition).^[^ ^53^ ^]^ However, a complete molecular picture of the inter‐domain interactions of PRD which contribute toward the stability of liquid‐like Htttex1 assemblies is currently unavailable.

To assess the effect of PRD on the structural ensemble of Httex1, we first performed PT‐WTE simulations of the N17‐Q_16_‐PRD monomer (Figure S12, Supporting Information). The ɑ‐helical fractions per‐residue (Figure S12A, Supporting Information) and mechanism of ɑ‐helical nucleation in Q_16_ are largely similar to N17‐Q_16_‐P_5_ (Figure S12B,C, Supporting Information). These observations collectively indicate that PRD does not impact the structural ensemble of N17‐Q_16_ region which is broadly consistent with ^13^C isotope‐labeling NMR experiments wherein the disruption of sequence connectivity between the PRD and Q_16_ tract (by introducing five glycine residues) only resulted in a modest increase in helicity of the latter.^[^ ^48^ ^]^ Further, the 2D‐intramolecular contact map of N17‐Q_16_‐PRD reveals that intradomain interactions of PRD which involve the polyproline P_10_ and P_11_ tracts are considerably more pronounced compared to its interdomain interactions with N17 and Q_16_ regions.

**Figure 5:**
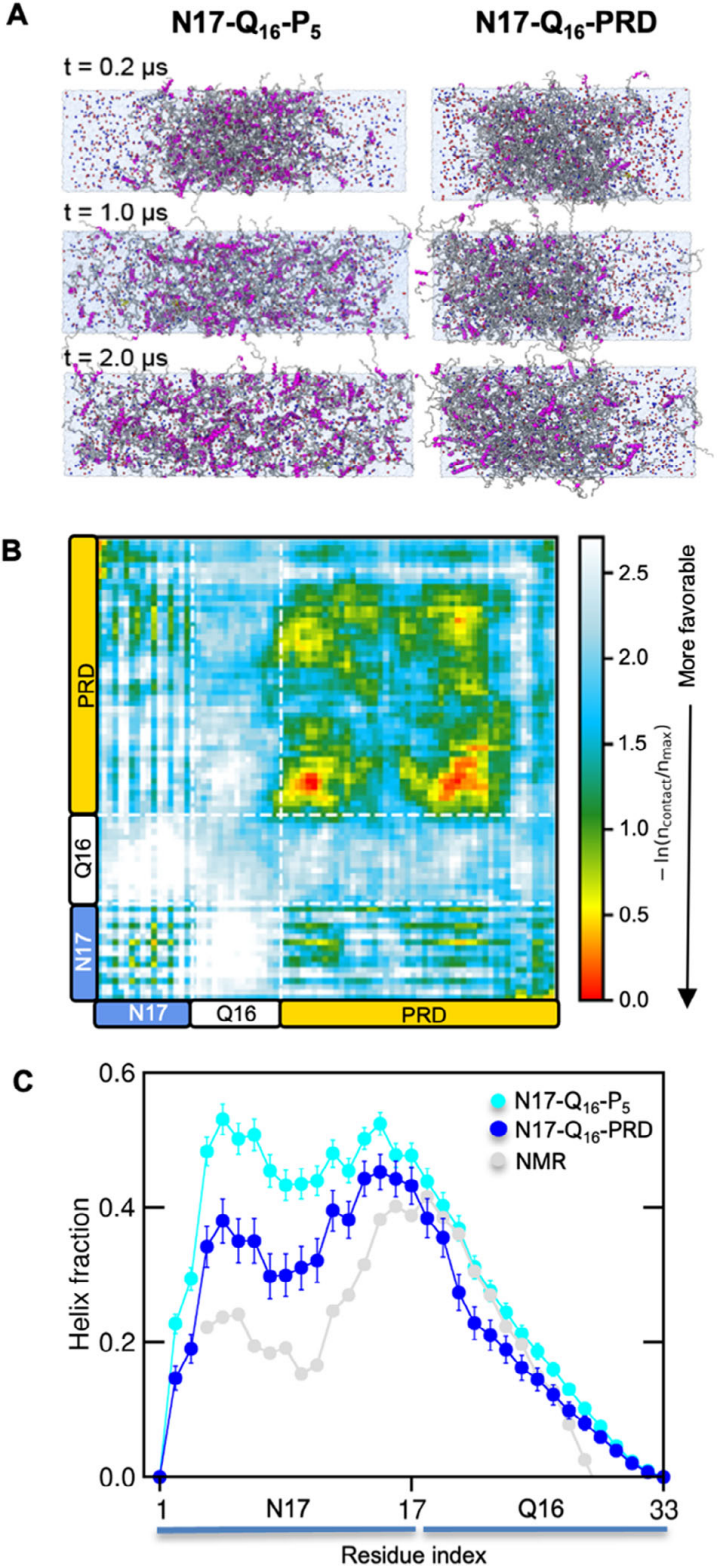
PRD‐mediated interactions stabilize the N17‐Q_16_‐PRD condensate and suppress N17 ɑ‐helicity. A) All‐atom MD simulation snapshots of N17‐Q_16_‐P_5_ (left) and N17‐Q_16_‐PRD (right) condensate systems over 2 µs of their respective trajectories. Protein chains are shown as ribbons and ions (Na^+^/Cl^−^) are shown as spheres. The snapshots indicate destabilization of the N17‐Q_16_‐P_5_ dense phase (0.2–1.0 µs) and its complete dissolution (2.0 µs), resulting in a homogeneous system. B) Pairwise 2D‐intermolecular contact map averaged over all chain pairs reveals significant contributions of the PRD toward stabilization of the N17‐Q_16_‐PRD. C) Mean fractional ɑ‐helicity per‐residue (computed over all dense phase chains) shows reduced ɑ‐helical formation within N17 for the N17‐Q_16_‐PRD condensate compared to a homogenous N17‐Q_16_‐P_5_ condensate system (i.e., lacking protein‐solvent interface). Error bars denote std. The error of mean overall chains.

To uncover the network of inter‐domain interactions implicated in the formation of Httex1 liquid‐like assemblies, we performed all‐atom simulations of pre‐formed N17‐Q_16_‐P_5_ and N17‐Q_16_‐PRD condensates (**Figure** **5**). The all‐atom condensates were constructed using the phase‐coexistence simulation method at coarse‐grained resolution and a subsequent backmapping strategy as described previously^[^ ^72^ ^]^ (also see Experimental Section). While the pre‐formed N17‐Q_16_‐PRD condensate remained stable over the course of the trajectory, N17‐Q_16_‐P_5_ failed to do so (Figure 5A). Mirroring the interactions observed at the single chain level, analysis of the 2D‐intramolecular contact map of N_17_‐Q_16_‐PRD condensate reveals that homotypic intermolecular interactions between PRD polyproline (P_10_/P_11_) tracts constitute the most significant type of intermolecular interaction (Figure 5B) which clearly highlights the requirement of PRD for condensate formation as seen in experiments. Similar to the case of N17‐Q_7/16_ dimers (Figure 4B), the ɑ‐helicity of the N17 N‐terminal region (aa:2‐11) is considerably elevated in the condensate compared to the isolated monomer due to enhanced intermolecular interactions within the crowded environment of the condensate (Figure S13A, Supporting Information). Further, we also observed the formation of transient intramolecular β‐sheets for a population of chains (Figure S13B, Supporting Information). However, the ɑ‐helicity of N17‐Q_16_‐PRD reduced compared to a homogenous N17‐Q_16_‐P_5_ condensate system which lacks a protein‐solvent interface (Figure 5C). This implies that N17/PRD interactions can suppress the ɑ‐helicity of N17 and potentially limit the aberrant interactions that promote liquid‐to‐solid phase transitions within Httex1 condensates.

## 3 Discussion

Emerging evidence from biophysical investigations suggests that there is a complex interplay between the N‐terminal and C‐terminal flanking sequence composition and structure, and polyQ length in modulating the overall aggregation propensity of polyQ proteins. Among the polyQ proteins implicated in neurodegenerative disorders, three of the proteins: Huntingtin exon 1 (Httex1), Androgen Receptor (AR), and Ataxin‐7 (Atx‐7) contain hydrophobic N‐terminal flanking sequences that induce and progressively increase the α‐helical propensity in the polyQ tract with length expansion.^[^ ^48^, ^73^, ^74^ ^]^ However, the increased α‐helical propensity in the polyQ region appears to have dissimilar effects on the aggregation of these polyQ‐containing sequences. While both AR and Atx‐7 showed suppressed polyQ aggregation for polyQ_25‐33_,^[^ ^73^, ^74^ ^]^ an aggregation of longer polyQ sequences (Q_46‐72_) is favored in the case of Httex1.^[^ ^55^ ^]^ It is therefore important to decipher the role of polyQ flanking sequences in order to obtain mechanistic insights into the amyloid aggregation of polyQ proteins and develop novel therapeutics to treat their associated‐neurodegenerative disorders.

The rapid aggregation kinetics of pathogenic Httex1 fragments limit the ability to achieve a complete atomic‐level characterization of the role of flanking domains on the structural and oligomerization landscape through experimental approaches alone. Further, conflicting results from previous MD simulation studies^[^ ^75^, ^76^, ^77^ ^]^ due to force field and water model inaccuracies have led to a lack of clarity regarding the precise molecular mechanisms of Httex1 oligomerization and pathogenic aggregation. Our study provides a comprehensive characterization of the structural ensembles of normal and pathogenic N17‐polyQ fragments using the AMBER03ws force field which was carefully tuned to achieve balanced secondary structure propensities and protein‐water interactions.^[^ ^47^, ^78^ ^]^ Importantly, our simulation ensembles correctly captured the length‐dependent increase in helical from Q_16_ to Q_46_, achieving good quantitative agreement with NMR in the case of N17‐Q_16/46_‐P_5_ fragments. Therefore, a distinguishing feature of this computational study is the extensive structural validation of N17‐Q_n_‐P_5_/PRD simulation ensembles against recent high‐resolution NMR experiments,^[^ ^48^, ^55^ ^]^ which considerably enhances the reliability of our predictions. In addition, our simulations also captured the effect of point mutations in the hydrophobic four‐residue motif (^14^LKSF^[^ ^17^ ^]^) which structurally couples N17 with the polyQ tract.^[^ ^48^ ^]^ The N17 domain is also subject to numerous post‐translational modifications (PTMs) which alter its α‐helical structure and impact the cellular localization, oligomerization, and aggregation of Htt.^[^ ^79^, ^80^, ^81^, ^82^ ^]^ Our chosen model and simulation methodology demonstrated here opens the way for accurate predictions of the effect of point mutations and PTMs on the structure and conformation of Httex1 and other polyQ proteins that contain N‐terminal hydrophobic motifs adjacent to the polyQ tract.

**Figure 6:**
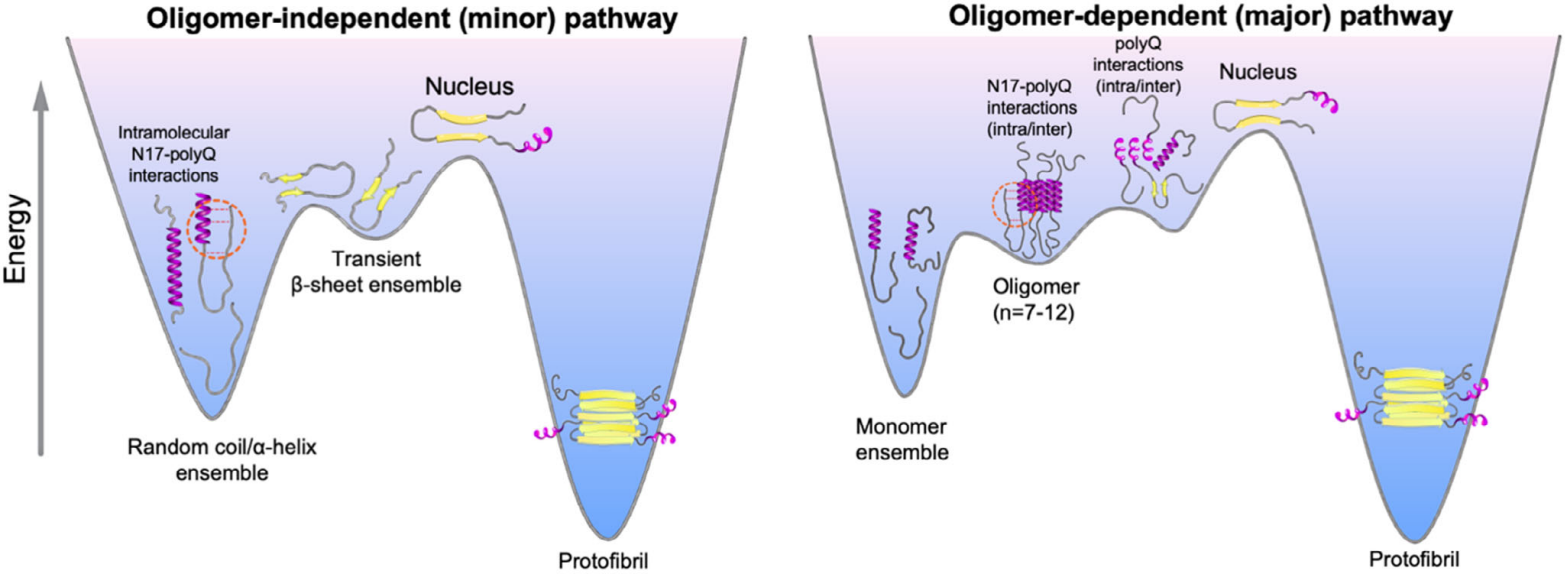
polyQ expansion promotes “domain crosstalk” which modulates the structural ensemble and oligomerization landscape of Httex1 to favor fibrillar aggregation. Schematic illustrating the effect of polyQ length expansion on the conformation and oligomerization of Httex1. polyQ expansion promotes inter‐domain N17/polyQ interactions. These interactions can (i) induce the emergence of transient β‐sheet conformers in monomers which may fold to form the critical nucleus and initiate protofibril formation (oligomer‐independent pathway), and (ii) counteract stabilization of N17‐mediated oligomers to favor polyQ/polyQ interactions which lead to nucleation and fibrillation. The nucleus size is shown as a monomer (n = 1) and is representative of Httex1‐Q_>23_.

Two opposing models are proposed to explain the coupling between structure and binding properties of polyQ with increasing length, namely the i) “linear lattice” model and, ii) “conformational emergence” model. The linear lattice model proposes that the enhanced multivalency (number of interaction sites) of polyglutamine upon length expansion coupled with the prevalence of an extended, random coil structural ensemble (abundant in N17‐Q_46_‐P_5_/Q_46_ simulations) its aberrant cellular interactions and increased binding affinity toward anti‐polyQ antibodies.^[^ ^83^, ^84^, ^85^ ^]^ Nevertheless, several studies also point toward the existence of β‐hairpin conformations formed in pathogenic polyQ^[^ ^17^ ^]^ and Httex1^[^ ^23^, ^65^, ^66^, ^86^ ^]^ in monomeric and oligomeric species. Interestingly, our results appear to reconcile the two opposing models in the case of pathogenic Httex1, by suggesting that the increased multivalency can also promote intramolecular interdomain (N17/polyQ) interactions which may give rise to “toxic” β‐conformations or those which proceed to form the aggregation‐competent nuclei more readily compared to disordered monomers (**Figure** **6**). Alternatively, such interactions may also promote polyQ “folding” to form the critical β‐hairpin nucleus, which can template the formation of amyloids through a kinetically competing N17‐independent pathway.^[^ ^33^ ^]^ Overall, our simulations directly detect the emergence of a conformational equilibrium between the predominant disordered conformer and potentially “toxic” β‐sheet conformers upon polyQ expansion, thereby providing a critical insight into polyQ aggregation and toxicity. It is important to note, however, that β‐conformers observed in our N17‐Q_32/46_‐P_5_ simulations constitute a lowly populated species (1‐2%), implying that such conformations are highly unfavorable relative to helical and coil conformations. These observations are qualitatively consistent with a highly unfavorable free energy of folding associated with polyglutamine nucleus formation (+12.2 kcal mol^−1^ for Q_47_).^[^ ^26^ ^]^ Alternatively, Thirumalai and co‐workers propose that such high energy conformers (referred to as N^*^ states) that occur on the conformational landscape of amyloidogenic sequences can provide an optimal route toward fibril assembly by initiating the formation of aggregation‐competent (i.e., “productive”) oligomers.^114^

We provide a high‐resolution view into the competing effects of domain “cross‐talk” and structural “transformation” on Httex1 oligomerization: i) interdomain N17/polyQ interactions destabilize oligomerization and, ii) α‐helical propagation into the polyQ tract which stabilizes oligomerization. These observations lead us to conclude that polyQ‐mediated interdomain interactions must outcompete the stabilizing effect of increased polyQ α‐helicity to favor nucleation and conversion of oligomers into amyloid aggregates. Our simulations of the Httex1 monomer and condensates uncover the significance of intermolecular PRD interactions in driving the formation of liquid‐like Httex1 assemblies. Further, N17/PRD intermolecular interactions which were shown to suppress N17 helicity could also limit the formation of aggregation‐competent oligomers and amyloid aggregates within Httex1 condensates. In conclusion, our findings support the idea that altering the balance of homo‐ (N17)^[^ ^87^ ^]^ and heterodomain (N17/polyQ and N17/PRD) interactions^[^ ^32^ ^]^ may serve as an effective therapeutic strategy for the inhibition of pathological self‐assembly of Httex1.

## 4 Experimental Section

### Initial Structure, Force Field Choice, and System Setup

For N17‐Q_16/24/32_‐P_5_ monomers, simulations were initiated from three different coil configurations generated based on the Flexible Meccano algorithm^[^ ^88^ ^]^ using the ProtSA webserver.^[^ ^89^ ^]^ For PT‐WTE simulations N17‐Q_46_‐P_5_, a single coil conformation was chosen among the three used for unbiased simulations. The three partially‐helical, initial conformations of N17‐Q_46_‐P_5_ were obtained from the first microsecond of a coil trajectory. For dimer simulations, the initial complex was extracted from the lowest energy NMR model of the N17 tetramer (PDB 6N8C). PolyQ sequences (Q_7/16_) were added to the monomer units of the N17 dimer using MODELLER.^[^ ^90^ ^]^ The initial configurations were solvated in octahedral boxes with 150 mm NaCl, and additional counter ions were added to achieve electroneutrality. The polypeptide and water topologies were described using parameters from the AMBER03ws force field^[^ ^47^ ^]^ (https://bitbucket.org/jeetain/all‐atom_ff_refinements/src/master/) and TIP4P/2005 water model^[^ ^91^ ^]^ respectively. For Na^+^ and Cl^−^ ions, modified LJ parameters^[^ ^92^ ^]^ were used to improve ion solubility. Details of box dimensions and duration of simulation trajectories for each monomer and dimer trajectories are provided in Table S1 (Supporting Information).

### Conventional MD Simulation Protocol

To remove any steric clashes, the solvated polypeptide systems were first relaxed by performing energy minimization using the steepest descent algorithm in GROMACS‐2020.4.^[^ ^93^ ^]^ Following minimization, the systems were simulated for 100 ps using a Nose‐Hoover thermostat^[^ ^94^ ^]^ with a coupling constant (τ_c_) of 1 ps to achieve temperature equilibration at 293 K. Next, a 100 ps simulation was conducted using the Berendsen barostat^[^ ^95^ ^]^ with isotropic coupling and a coupling constant (τ_p_) of 5 ps for pressure control, to achieve pressure equilibration at 1 bar. Production simulations were performed in the NVT ensemble was performed using the Langevin Middle Integrator^[^ ^96^ ^]^ (friction coefficient = 1 ps^−1^) in OpenMM‐7.5.^[^ ^97^ ^]^ Short‐range nonbonded interactions were computed using a cutoff radius of 0.9 nm, while long‐range electrostatics were described with the PME method.^[^ ^98^, ^99^ ^]^ The hydrogen masses were increased by 1.5 times to enable a simulation timestep of 4 fs^[^ ^100^ ^]^ for the production runs, and hydrogen‐containing bonds were constrained using the SHAKE algorithm.^[^ ^101^ ^]^

### Parallel Tempering Well‐Tempered Ensemble (PT‐WTE) MD Simulation Protocol and Assessment of Convergence

Following energy minimization, temperature, and pressure equilibration of the system at 293 K and 1 bar, temperature replicas were prepared and equilibrated in the NVT ensemble without exchange for 10 ns at higher temperatures using the Langevin integrator (friction coefficient = 1 ps^−1^). A total of 16 temperature replicas were generated based on a geometric progression that spanned a range from 293 to 500 K. Long‐range electrostatic interactions were modeled using the Particle Mesh Ewald method^[^ ^98^, ^99^ ^]^ with a real space distance cutoff of 0.9 nm. The hydrogen mass repartitioning scheme^[^ ^100^ ^]^ was used to reduce the computational cost along the LINCS algorithm^[^ ^102^ ^]^ which was used to constrain all bonds, allowing a time step of 5 fs. Following temperature equilibration of the replicas, production runs were performed in the NVT ensemble with bias potentials (bias factor = 72 for N17‐Q_46_‐P_5_, 96 for N17‐Q_16_‐PRD) deposited every 4 ps to increase fluctuations in the system potential energy (collective variable) resulting in a well‐temperature ensemble (WTE) with improved frequency of conformational exchange between adjacent replicas,^[^ ^103^, ^104^ ^]^ which was attempted every 1 ps. The initial height and width of the Gaussian potential were set to 1.5 and 456.0 kJ mol^−1^. Exchange probabilities between adjacent replicas varied from 17–30%.

For N17‐Q_46_‐P_5_, the first 150 ns from the 293 K replica was discarded as equilibration time respectively, during which the initial height of the gaussian potential dropped below 0.25 kJ mol^−1^, and sufficient overlap in the potential energy distributions between adjacent temperature replicas was observed. The initial configurations for each replica underwent several round trips through the entire temperature space, which indicates efficient conformational exchange between replicas. The convergence of the 293 K replica trajectory was assessed by computing the radius of gyration (R_g_) and ɑ‐helical fraction over independent blocks of the trajectory (Figure S4C,D, Supporting Information).

### Bayesian‐inspired Monte Carlo Sampling

To assess the relative timing of helix formation between different regions, a Bayesian‐inspired Monte Carlo sampling approach was employed. Average initiation times and standard errors were first computed for each residue based on the simulation data. For each residue, the first frame (or corresponding time in nanoseconds) when it adopted an α‐helical conformation is recorded, defining this as the residue's “initiation time.” Residues that never adopted a helical conformation throughout the simulation were marked as having undefined initiation times. Each residue's initiation time was assumed to follow a normal distribution centered around its mean with the corresponding standard error, accounting for measurement and analysis uncertainties.

For statistical sampling, 1 000 000 Monte Carlo trials were performed. In each trial, a random sample was drawn from the normal distribution of each residue's initiation time. The sampled initiation times were then averaged within defined regions, such as the central and polyQ regions. To estimate the probability that helix formation occurs earlier in the central region, the sampled averages of the two regions were compared across all trials. The fraction of trials in which the central region exhibited a lower average initiation time than the polyQ region provided an estimate of P(central < polyQ), quantifying the likelihood that helix formation initiates in the central region before propagating into the polyQ tract.

### All‐Atom Condensate Simulations

All‐atom dense‐phase simulations of N17‐Q_16_‐P_5_ and N17‐Q_16_‐PRD were prepared using the method previously.^[^ ^72^ ^]^ Initial structures for multichain simulations were generated by equilibrating 170 chains of N17‐Q_16_‐P_5_ at 200 K and 90 chains of N17‐Q_16_‐PRD at 300 K, using coarse‐grained (CG) coexistence simulations with the HPS‐SS model.^[^ ^105^, ^106^ ^]^ Notably, N17‐Q_16_‐P_5_ was unable to form a stable condensate at 300 K under these simulation conditions. Subsequently, the CG dense phase configurations in the slab geometry were used to reconstruct all‐atom slab configurations with Modeller.^[^ ^90^, ^107^ ^]^ Potential steric clashes were resolved through short molecular dynamics simulations with OpenMM‐7.6^[^ ^97^ ^]^ employing the AMBER03^[^ ^108^ ^]^ force field and OBC implicit solvent model.^[^ ^109^ ^]^ The resulting relaxed protein systems were then subjected to equilibration and production simulations as described in the preceding sections. The resulting relaxed protein systems were then subjected to equilibration in Gromacs as described in the preceding sections. The Gromacs files were converted to Amber inputs using the “gromber” function in Parmed^[^ ^110^ ^]^ and hydrogen mass repartitioning^[^ ^100^ ^]^ to 1.5 amu was performed during conversion to facilitate a 4 fs time step. Production simulations were performed using the AMBER22 simulation package.

### Trajectory Analysis

Secondary structure calculations were performed based on the DSSP library^[^ ^111^ ^]^ using the gmx do_dssp program. The calculation of NMR chemical shifts and comparison with experiments was performed using SPARTA+ as described in our previous study.^[^ ^112^ ^]^ The calculation of secondary structure (SS) maps for assessing the α‐helicity cooperativity of N17‐polyQ regions was performed using a modified version of the python script provided as part of the original study to include the DSSP definition for identification of α‐helices. For the detection of bifurcated hydrogen bonds (B‐HBs), gmx hbond in combination with in‐house python scripts were used to compute the fraction of trajectory frames in which glutamine sidechain to the backbone (i → i‐4) hydrogen bonds coexisted with backbone‐backbone (i‐4 → i) hydrogen bonds. B‐HB analysis was carried out only on frames in which >30% of the residues adopted an α‐helical conformation. For the analysis and visualization of β‐sheet conformations, only frames where >4 residues adopted a β‐sheet conformations were extracted from unbiased and PT‐WTE trajectories. For contact analysis, two residues i and j with sequence separation greater than 3 (|i‐j|>3) were in contact if any two heavy atoms of the residues were within 0.6 nm of each other. The chosen distance cutoff has been extensively tested in our previous studies^[^ ^72^, ^112^, ^113^ ^]^ and is large enough to include the diverse contact modes such as hydrogen bonds (<0.35 nm), van der Waal interactions, and salt‐bridges (<0.6 nm). For the analysis of N17‐polyQ dimerization, the minimum distance, and order parameters (number of N17‐N17 contacts and inter‐helical angle) for 2D‐PMFs (Figure 3C) were computed using gmx mindist and gmx gangle.

## Conflict of Interest

The authors declare no conflict of interest.

## Supporting information



Supporting Information

## Data Availability

The data that support the findings of this study are available from the corresponding author upon reasonable request.;

## References

[1] C. A. P. P Ross , Neuron 2002, 35, 819. 12372277 10.1016/s0896-6273(02)00872-3

[2] J. Shao , M. I. Diamond , Hum. Mol. Genet. 2007, 16, R115. 17911155 10.1093/hmg/ddm213

[3] A. Adegbuyiro , F. Sedighi , A. W. Pilkington , S. Groover , J. Legleiter , Biochemistry 2017, 56, 1199. 28170216 10.1021/acs.biochem.6b00936 PMC5727916

[4] M. DiFiglia , E. Sapp , K. O. Chase , S. W. Davies , G. P. Bates , J. P. Vonsattel , N. Aronin , Science 1997, 277, 1990. 9302293 10.1126/science.277.5334.1990

[5] K. A. Sieradzan , A. O. Mechan , L. Jones , E. E. Wanker , N. Nukina , D. M. A. Mann , Exp. Neurol. 1999, 156, 92. 10192780 10.1006/exnr.1998.7005

[6] S. J. Tabrizi , M. D. Flower , C. A. Ross , E. J. H Wild , Nat. Rev. Neurol. 2020, 16, 529. 32796930 10.1038/s41582-020-0389-4

[7] C. A. Ross , E. H. Aylward , E. J. Wild , D. R. Langbehn , J. D. Long , J. H. Warner , R. I. Scahill , B. R. Leavitt , J. C. Stout , J. S. Paulsen , R. Reilmann , P. G. Unschuld , A. Wexler , R. L. Margolis , S. J. H D: N H Tabrizi , Nat. Rev. Neurol. 2014, 10, 204. 24614516 10.1038/nrneurol.2014.24

[8] G. P. Bates , R. Dorsey , J. F. Gusella , M. R. Hayden , C. Kay , B. R. Leavitt , M. Nance , C. A. Ross , R. I. Scahill , R. Wetzel , E. J. Wild , S. J. H D Tabrizi , Nat. Rev. Dis. Primers 2015, 1, 15005. 27188817 10.1038/nrdp.2015.5

[9] S. Gunawardena , L.‐S. Her , R. G. Brusch , R. A. Laymon , I. R. Niesman , B. Gordesky‐Gold , L. Sintasath , N. M. Bonini , L. S. B. Goldstein , Neuron 2003, 40, 25. 14527431 10.1016/s0896-6273(03)00594-4

[10] L. Li , H. Liu , P. Dong , D. Li , W. R. Legant , J. B. Grimm , L. D. Lavis , E. Betzig , R. Tjian , Z. Liu , Elife 2016, 5, 1. 10.7554/eLife.17056 PMC4972539 27484239

[11] M. Duyao , C. Ambrose , R. Myers , A. Novelletto , F. Persichetti , M. Frontali , S. Folstein , C. Ross , M. Franz , M. Abbott , J. Gray , P. Conneally , A. Young , J. Penney , Z. Hollingsworth , I. Shoulson , A. Lazzarini , A. Falek , W. Koroshetz , D. Sax , E. Bird , J. Vonsattel , E. Bonilla , J. Alvir , J. Bickham Conde , J.‐H. Cha , L. Dure , F. Gomez , M. Ramos , J. Sanchez‐Ramos , et al., Nat. Genet. 1993, 4, 387. 8401587 10.1038/ng0893-387

[12] M. Kim , H.‐S. Lee , G. LaForet , C. McIntyre , E. J. Martin , P. Chang , T. W. Kim , M. Williams , P. H. Reddy , D. Tagle , F. M. Boyce , L. Won , A. Heller , N. Aronin , M. DiFiglia , J. Neurosci. 1999, 19, 964. 9920660 10.1523/JNEUROSCI.19-03-00964.1999 PMC6782141

[13] A. Yamamoto , J. J. Lucas , R. Hen , Cell 2000, 101, 57. 10778856 10.1016/S0092-8674(00)80623-6

[14] H. Yang , S. Yang , L. Jing , L. Huang , L. Chen , X. Zhao , W. Yang , Y. Pan , P. Yin , Z. S. Qin , B. Tang , S. Li , X.‐J. Li , Nat. Commun. 2020, 11, 2582. 32444599 10.1038/s41467-020-16318-1 PMC7244548

[15] J. B. Warner , K. M. Ruff , P. S. Tan , E. A. Lemke , R. V. Pappu , H. A. Lashuel , J. Am. Chem. Soc. 2017, 139, 14456. 28937758 10.1021/jacs.7b06659 PMC5677759

[16] S. L. Crick , M. Jayaraman , C. Frieden , R. Wetzel , R. V. Pappu , Proc. Natl. Acad. Sci. USA 2006, 103, 16764. 17075061 10.1073/pnas.0608175103 PMC1629004

[17] Y. Nagai , T. Inui , H. A. Popiel , N. Fujikake , K. Hasegawa , Y. Urade , Y. Goto , H. Naiki , T. Toda , Nat. Struct. Mol. Biol. 2007, 14, 332. 17369839 10.1038/nsmb1215

[18] I. Sánchez , C. Mahlke , J. Yuan , Nature 2003, 421, 373. 12540902 10.1038/nature01301

[19] S. L. Crick , K. M. Ruff , K. Garai , C. Frieden , R. V. Pappu , Proc. Natl. Acad. Sci. USA 2013, 110, 20075. 24282292 10.1073/pnas.1320626110 PMC3864320

[20] G. Cisbani , F. Cicchetti , Cell Death Dis. 2012, 3, 382. 10.1038/cddis.2012.121 PMC3434668 22932724

[21] I. Matlahov , P. C. van der Wel , Exp. Biol. Med. 2019, 244, 1584. 10.1177/1535370219856620 PMC6920524 31203656

[22] D. M. Hatters , IUBMB Life 2008, 60, 724. 18756529 10.1002/iub.111

[23] K. Shen , B. Calamini , J. A. Fauerbach , B. Ma , S. H. Shahmoradian , I. L. Serrano Lachapel , W. Chiu , D. C. Lo , J. Frydman , Elife 2016, 5, 18065. 10.7554/eLife.18065 PMC5135392 27751235

[24] T. T. M. Phan , J. D. Schmit , Biophys. J. 2020, 118, 2989. 32497516 10.1016/j.bpj.2020.05.013 PMC7300330

[25] M. Chen , P. G. Wolynes , Proc. Natl. Acad. Sci. USA 2017, 114, 4406. 28400517 10.1073/pnas.1702237114 PMC5410817

[26] A. M. Bhattacharyya , A. K. Thakur , R. Wetzel , Proc. Natl. Acad. Sci. USA 2005, 102, 15400. 16230628 10.1073/pnas.0501651102 PMC1266079

[27] K. Kar , M. Jayaraman , B. Sahoo , R. Kodali , R. Wetzel , Nat. Struct. Mol. Biol. 2011, 18, 328. 21317897 10.1038/nsmb.1992 PMC3075957

[28] M. Chen , M. Tsai , W. Zheng , P. G. Wolynes , J. Am. Chem. Soc. 2016, 138, 15197. 27786478 10.1021/jacs.6b08665 PMC5803750

[29] T. M. Phan , J. D. Schmit , Biophys. J. 2022, 121, 2931. 35778843 10.1016/j.bpj.2022.06.031 PMC9388551

[30] T. Kandola , S. Venkatesan , J. Zhang , B. T. Lerbakken , A. Von Schulze , J. F. Blanck , J. Wu , J. R. Unruh , P. Berry , J. J. Lange , A. C. Box , M. Cook , C. Sagui , R. Halfmann , Elife 2023, 12, 1. 10.7554/eLife.86939 PMC10624427 37921648

[31] A. K. Thakur , M. Jayaraman , R. Mishra , M. Thakur , V. M. Chellgren , I.‐J. L Byeon , D. H. Anjum , R. Kodali , T. P. Creamer , J. F. Conway , A. M Gronenborn , R. Wetzel , Nat. Struct. Mol. Biol. 2009, 16, 380. 19270701 10.1038/nsmb.1570 PMC2706102

[32] S. Tam , C. Spiess , W. Auyeung , L. Joachimiak , B. Chen , M. A. Poirier , J. Frydman , Nat. Struct. Mol. Biol. 2009, 16, 1279. 19915590 10.1038/nsmb.1700 PMC2788664

[33] M. Jayaraman , R. Mishra , R. Kodali , A. K. Thakur , L. M. I. Koharudin , A. M. Gronenborn , R. Wetzel , Biochemistry 2012, 51, 2706. 22432740 10.1021/bi3000929 PMC3394396

[34] V. N. Sivanandam , M. Jayaraman , C. L. Hoop , R. Kodali , R. Wetzel , P. C. A. van der Wel , J. Am. Chem. Soc. 2011, 133, 4558. 21381744 10.1021/ja110715f PMC3109494

[35] C. L. Hoop , H.‐K. Lin , K. Kar , G. Magyarfalvi , J. M. Lamley , J. C. Boatz , A. Mandal , J. R. Lewandowski , R. Wetzel , P. C. A. van der Wel , Proc. Natl. Acad. Sci. USA 2016, 113, 1546. 26831073 10.1073/pnas.1521933113 PMC4760812

[36] P. C. A. van der Wel , Biochem. Soc. Trans. 2024, 52, 719. 38563485 10.1042/BST20230731 PMC11088915

[37] B. Kokona , Z. P. Rosenthal , R. Fairman , Biochemistry 2014, 53, 6738. 25310851 10.1021/bi500449a

[38] L. G. Nucifora , K. A. Burke , X. Feng , N. Arbez , S. Zhu , J. Miller , G. Yang , T. Ratovitski , M. Delannoy , P. J. Muchowski , S. Finkbeiner , J. Legleiter , C. A. Ross , M. A. Poirier , J. Biol. Chem. 2012, 287, 16017. 22433867 10.1074/jbc.M111.252577 PMC3346083

[39] S. A. Kotler , V. Tugarinov , T. Schmidt , A. Ceccon , D. S. Libich , R. Ghirlando , C. D. Schwieters , G. M. Clore , Proc. Natl. Acad. Sci. USA 2019, 116, 3562. 30808748 10.1073/pnas.1821216116 PMC6397591

[40] A. Ceccon , V. Tugarinov , F. Torricella , G. M. Clore , Proc. Natl. Acad. Sci. USA 2022, 119, 2207690119. 10.1073/pnas.2207690119 PMC9303973 35858329

[41] S. N. Moldovean , V. Chiş , ACS Chem. Neurosci. 2020, 11, 105. 31841621 10.1021/acschemneuro.9b00561

[42] K. Lindorff‐Larsen , P. Maragakis , S. Piana , M. P. Eastwood , R. O. Dror , D. E. Shaw , PLoS One 2012, 7, 32131. 10.1371/journal.pone.0032131 PMC3285199 22384157

[43] R. B. Best , N.‐V. Buchete , G. Hummer , Biophys. J. 2008, 95, L07. 18456823 10.1529/biophysj.108.132696 PMC2426634

[44] J. Mittal , R. B. Best , Biophys. J. 2010, 99, L26. 20682244 10.1016/j.bpj.2010.05.005 PMC2913180

[45] S. Rauscher , V. Gapsys , M. J. Gajda , M. Zweckstetter , B. L. De Groot , J. Chem. Theory Comput. 2015, 11, 5513. 26574339 10.1021/acs.jctc.5b00736

[46] J. Henriques , C. Cragnell , M. Skepö , J. Chem. Theory Comput. 2015, 11, 3420. 26575776 10.1021/ct501178z

[47] R. B. Best , W. Zheng , J. Mittal , J. Chem. Theory Comput. 2014, 10, 5113. 25400522 10.1021/ct500569b PMC4230380

[48] A. Urbanek , M. Popovic , A. Morató , A. Estaña , C. A. Elena‐Real , P. Mier , A. Fournet , F. Allemand , S. Delbecq , M. A. Andrade‐Navarro , J. Cortés , N. Sibille , P. Bernadó , Structure 2020, 28, 733. 32402249 10.1016/j.str.2020.04.008

[49] C. A. Elena‐Real , A. Sagar , A. Urbanek , M. Popovic , A. Morató , A. Estaña , A. Fournet , C. Doucet , X. L. Lund , Z.‐D. Shi , L. Costa , A. Thureau , F. Allemand , R. E. Swenson , P.‐E. Milhiet , R. Crehuet , A. Barducci , J. Cortés , D. Sinnaeve , N. Sibille , P. Bernadó , Nat. Struct. Mol. Biol. 2023, 30, 309. 36864173 10.1038/s41594-023-00920-0

[50] P. Mohanty , U. Kapoor , D. Sundaravadivelu Devarajan , T. M. Phan , A. Rizuan , J. Mittal , Biochemistry 2022, 22, 2443. 10.1021/acs.biochem.2c00210 PMC9669140 35802394

[51] S. F. Banani , H. O. Lee , A. A. Hyman , M. K. B C: O C B Rosen , Nat. Rev. Mol. Cell Biol. 2017, 18, 285. 28225081 10.1038/nrm.2017.7 PMC7434221

[52] G. L. Dignon , R. B. Best , J. Mittal , Annu. Rev. Phys. Chem. 2020, 71, 53. 32312191 10.1146/annurev-physchem-071819-113553 PMC7469089

[53] T. R. Peskett , F. Rau , J. O'Driscoll , R. Patani , A. R. Lowe , H. R. Saibil , Mol. Cell 2018, 70, 588. 29754822 10.1016/j.molcel.2018.04.007 PMC5971205

[54] B. Szała‐Mendyk , T. M. Phan , P. Mohanty , J. Mittal , Curr. Opin. Chem. Biol. 2023, 75, 102333. 37267850 10.1016/j.cbpa.2023.102333 PMC10527940

[55] C. A. Elena‐Real , A. Urbanek , X. L. Lund , A. Morató , A. Sagar , A. Fournet , A. Estaña , T. Bellande , F. Allemand , J. Cortés , N. Sibille , R. Melki , P. Bernadó , Structure 2023, 31, 644. 37119819 10.1016/j.str.2023.04.003

[56] G. H. Zerze , W. Zheng , R. B. Best , J. Mittal , J. Phys. Chem. Lett. 2019, 10, 2227. 30990694 10.1021/acs.jpclett.9b00850 PMC7507668

[57] P. Robustelli , S. Piana , D. E. Shaw , Proc. Natl. Acad. Sci. USA 2018, 115, E4758. 29735687 10.1073/pnas.1800690115 PMC6003505

[58] M. Jayaraman , R. Kodali , B. Sahoo , A. K. Thakur , A. Mayasundari , R. Mishra , C. B. Peterson , R. Wetzel , J. Mol. Biol. 2012, 415, 881. 22178474 10.1016/j.jmb.2011.12.010 PMC3568928

[59] B. Sahoo , D. Singer , R. Kodali , T. Zuchner , R. Wetzel , Biochemistry 2014, 53, 3897. 24921664 10.1021/bi500300c PMC4075985

[60] J. A. Marsh , V. K. Singh , Z. Jia , J. D. Forman‐Kay , Protein Sci. 2006, 15, 2795. 17088319 10.1110/ps.062465306 PMC2242444

[61] A. Escobedo , B. Topal , M. B. A. Kunze , J. Aranda , G. Chiesa , D. Mungianu , G. Bernardo‐Seisdedos , B. Eftekharzadeh , M. Gairí , R. Pierattelli , I. C. Felli , T. Diercks , O. Millet , J. García , M. Orozco , R. Crehuet , K. Lindorff‐Larsen , X. Salvatella , Nat. Commun. 2019, 10, 2034. 31048691 10.1038/s41467-019-09923-2 PMC6497633

[62] J. Iglesias , M. Sanchez‐Martínez , R. SS‐M Crehuet , Intrinsically Disord. Proteins 2013, 1, 25323. 10.4161/idp.25323 PMC5424797 28516013

[63] W. S. Tang , N. L. Fawzi , J. Mittal , J. Phys. Chem. B 2020, 124, 9505. 33078950 10.1021/acs.jpcb.0c07545 PMC7880584

[64] J. Mittal , T. Phan , P. Mohanty , Res. Sq. 2025.

[65] J. Miller , M. Arrasate , E. Brooks , C. P. Libeu , J. Legleiter , D. Hatters , J. Curtis , K. Cheung , P. Krishnan , S. Mitra , K. Widjaja , B. A. Shaby , G. P. Lotz , Y. Newhouse , E. J. Mitchell , A. Osmand , M. Gray , V. Thulasiramin , F. Saudou , M. Segal , X. W. Yang , E. Masliah , L. M. Thompson , P. J. Muchowski , K. H. Weisgraber , S. Finkbeiner , Nat. Chem. Biol. 2011, 7, 925. 22037470 10.1038/nchembio.694 PMC3271120

[66] C. Peters‐Libeu , J. Miller , E. Rutenber , Y. Newhouse , P. Krishnan , K. Cheung , D. Hatters , E. Brooks , K. Widjaja , T. Tran , S. Mitra , M. Arrasate , L. A. Mosquera , D. Taylor , K. H. Weisgraber , S. Finkbeiner , J. Mol. Biol. 2012, 421, 587. 22306738 10.1016/j.jmb.2012.01.034 PMC3358578

[67] K. Kar , C. L. Hoop , K. W. Drombosky , M. A. Baker , R. Kodali , I. Arduini , P. C. A. van der Wel , W. S. Horne , R. Wetzel , J. Mol. Biol. 2013, 425, 1183. 23353826 10.1016/j.jmb.2013.01.016 PMC3602386

[68] J. M. Bravo‐Arredondo , N. C. Kegulian , T. Schmidt , N. K. Pandey , A. J. Situ , T. S. Ulmer , R. Langen , J. Biol. Chem. 2018, 293, 19613. 30315108 10.1074/jbc.RA118.004808 PMC6314148

[69] N. S. Caron , C. R. Desmond , J. Xia , R. Truant , Proc. Natl. Acad. Sci. USA 2013, 110, 14610. 23898200 10.1073/pnas.1301342110 PMC3767516

[70] D. De Sancho , R. B. Best , J. Am. Chem. Soc. 2011, 133, 6809. 21480610 10.1021/ja200834s

[71] N. K. Pandey , J. Varkey , A. Ajayan , G. George , J. Chen , R. Langen , J. Biol. Chem. 2024, 300, 105585. 38141760 10.1016/j.jbc.2023.105585 PMC10825056

[72] W. Zheng , G. L. Dignon , N. Jovic , X. Xu , R. M. Regy , N. L. Fawzi , Y. C. Kim , R. B. Best , J. Mittal , J. Phys. Chem. B 2020, 124, 11671. 33302617 10.1021/acs.jpcb.0c10489 PMC7879053

[73] B. Eftekharzadeh , A. Piai , G. Chiesa , D. Mungianu , J. García , R. Pierattelli , I. C. Felli , X. Salvatella , Biophys. J. 2016, 110, 2361. 27276254 10.1016/j.bpj.2016.04.022 PMC4900447

[74] J.‐Y. Hong , D.‐D. Wang , W. Xue , H.‐W. Yue , H. Yang , L.‐L. Jiang , W.‐N. Wang , H.‐Y. Hu , Sci. Rep. 2019, 9, 7481. 31097749 10.1038/s41598-019-43926-9 PMC6522498

[75] M. Długosz , J. Trylska , J. Phys. Chem. B 2011, 115, 11597. 21910495 10.1021/jp206373g

[76] H. Kang , F. X. Vázquez , L. Zhang , P. Das , L. Toledo‐Sherman , B. Luan , M. Levitt , R. Zhou , J. Am. Chem. Soc. 2017, 139, 8820. 28609090 10.1021/jacs.7b00838 PMC5835228

[77] M. Khaled , B. Strodel , A. Sayyed‐Ahmad , Front. Mol. Biosci. 2023, 10, 1. 10.3389/fmolb.2023.1143353 PMC10123271 37101557

[78] R. B. Best , J. Mittal , J. Phys. Chem. B 2010, 114, 14916. 21038907 10.1021/jp108618d

[79] S. M. DeGuire , F. S. Ruggeri , M.‐B. Fares , A. Chiki , U. Cendrowska , G. Dietler , H. A. Lashuel , J. Biol. Chem. 2018, 293, 18540. 30185623 10.1074/jbc.RA118.004621 PMC6290154

[80] L. F. DiGiovanni , A. J. Mocle , J. Xia , R. Truant , Hum. Mol. Genet. 2016, 25, 3937. 27466181 10.1093/hmg/ddw234 PMC5291230

[81] X. Gu , J. P. Cantle , E. R. Greiner , C. Y. D. Lee , A. M. Barth , F. Gao , C. S. Park , Z. Zhang , S. Sandoval‐Miller , R. L. Zhang , M. Diamond , I. Mody , G. Coppola , X. W. Yang , Neuron 2015, 85, 726. 25661181 10.1016/j.neuron.2015.01.008 PMC4386927

[82] R. Mishra , C. L. Hoop , R. Kodali , B. Sahoo , P. C. A. van der Wel , R. Wetzel , J. Mol. Biol. 2012, 424, 1. 22999956 10.1016/j.jmb.2012.09.011 PMC3488119

[83] M. J. Bennett , K. E. Huey‐Tubman , A. B. Herr , A. P. West , S. A. Ross , P. J. A Bjorkman , Proc. Natl. Acad. Sci. USA 2002, 99, 11634. 12193654 10.1073/pnas.182393899 PMC129321

[84] G. E. Owens , D. M. New , A. P. West , P. J. Bjorkman , J. Mol. Biol. 2015, 427, 2507. 26047735 10.1016/j.jmb.2015.05.023 PMC4520773

[85] F. A. C. Klein , G. Zeder‐Lutz , A. Cousido‐Siah , A. Mitschler , A. Katz , P. Eberling , J.‐L. Mandel , A. Podjarny , Y. Trottier , Hum. Mol. Genet. 2013, 22, 4215. 23777629 10.1093/hmg/ddt273

[86] M. Kim , Prion 2013, 7, 221. 23370273 10.4161/pri.23807 PMC3783107

[87] R. Mishra , M. Jayaraman , B. P. Roland , E. Landrum , T. Fullam , R. Kodali , A. K. Thakur , I. Arduini , R. Wetzel , J. Mol. Biol. 2012, 415, 900. 22178478 10.1016/j.jmb.2011.12.011 PMC3267848

[88] V. Ozenne , F. Bauer , L. Salmon , J.‐R. Huang , M. R. Jensen , S. Segard , P. Bernado , C. Charavay , M. Blackledge , Bioinformatics 2012, 28, 1463. 22613562 10.1093/bioinformatics/bts172

[89] J. Estrada , P. Bernadó , M. Blackledge , J. Sancho , BMC Bioinformatics 2009, 10, 104. 19356231 10.1186/1471-2105-10-104 PMC2674053

[90] B. Webb , A. Sali , Curr. Protoc. Bioinformatics 2016, 54, 5.6.1. 10.1002/cpbi.3 PMC5031415 27322406

[91] J. L. F. Abascal , C. Vega , J. Chem. Phys. 2005, 123, 234505. 16392929 10.1063/1.2121687

[92] Y. Luo , B. Roux , J. Phys. Chem. Lett. 2010, 1, 183.

[93] S. Páll , A. Zhmurov , P. Bauer , M. Abraham , M. Lundborg , A. Gray , B. Hess , E. Lindahl , J. Chem. Phys. 2020, 153, 134110. 33032406 10.1063/5.0018516

[94] D. J. Evans , B. L. Holian , J. Chem. Phys. 1985, 83, 4069.

[95] H. J. C. Berendsen , J. P. M. Postma , W. F. van Gunsteren , A. DiNola , J. R. Haak , J. Chem. Phys. 1984, 81, 3684.

[96] Z. Zhang , X. Liu , K. Yan , M. E. Tuckerman , J. Liu , J. Phys. Chem. A 2019, 123, 6056. 31117592 10.1021/acs.jpca.9b02771

[97] P. Eastman , J. Swails , J. D. Chodera , R. T. McGibbon , Y. Zhao , K. A. Beauchamp , L.‐P. Wang , A. C. Simmonett , M. P. Harrigan , C. D. Stern , R. P. Wiewiora , B. R. Brooks , V. S. Pande , PLoS Comput. Biol. 2017, 13, 1005659. 10.1371/journal.pcbi.1005659 PMC5549999 28746339

[98] T. Darden , D. York , L. Pedersen , J. Chem. Phys. 1993, 98, 10089.

[99] U. Essmann , L. Perera , M. L. Berkowitz , T. Darden , H. Lee , L. G. Pedersen , J. Chem. Phys. 1995, 103, 8577.

[100] C. W. Hopkins , S. Le Grand , R. C. Walker , A. E. Roitberg , J. Chem. Theory Comput. 2015, 11, 1864. 26574392 10.1021/ct5010406

[101] J.‐P. Ryckaert , G. Ciccotti , H. J. C. Berendsen , J. Comput. Phys. 1977, 23, 327.

[102] B. P‐L Hess , J. Chem. Theory Comput. 2008, 4, 116. 26619985 10.1021/ct700200b

[103] M. Bonomi , M. Parrinello , Phys. Rev. Lett. 2010, 104, 190601. 20866953 10.1103/PhysRevLett.104.190601

[104] M. Deighan , M. Bonomi , J. Pfaendtner , J. Chem. Theory Comput. 2012, 8, 2189. 26588950 10.1021/ct300297t

[105] R. M. Regy , J. Thompson , Y. C. Kim , J. Mittal , Protein Sci. 2021, 30, 1371. 33934416 10.1002/pro.4094 PMC8197430

[106] A. Rizuan , N. Jovic , T. M. Phan , Y. C. Kim , J. Mittal , J. Chem. Inf. Model. 2022, 62, 4474. 36066390 10.1021/acs.jcim.2c00450 PMC10165611

[107] A. Fiser , A. Šali , Methods Enzymol. 2003, 374, 461. 14696385 10.1016/S0076-6879(03)74020-8

[108] Y. Duan , C. Wu , S. Chowdhury , M. C. Lee , G. Xiong , W. Zhang , R. Yang , P. Cieplak , R. Luo , T. Lee , J. Caldwell , J. Wang , P. Kollman , J. Comput. Chem. 2003, 24, 1999. 14531054 10.1002/jcc.10349

[109] A. Onufriev , D. Bashford , D. A. Case , J. Phys. Chem. B 2000, 104, 3712.

[110] M. R. Shirts , C. Klein , J. M. Swails , J. Yin , M. K. Gilson , D. L. Mobley , D. A. Case , E. D. Zhong , J. Comput. Aided Mol. Des. 2017, 31, 147. 27787702 10.1007/s10822-016-9977-1 PMC5581938

[111] W. Kabsch , C. Sander , Biopolymers 1983, 22, 2577. 6667333 10.1002/bip.360221211

[112] P. Mohanty , J. Shenoy , A. Rizuan , J. F. Mercado‐Ortiz , N. L. Fawzi , J. Mittal , Proc. Natl. Acad. Sci. USA 2023, 120, 2305625120. 10.1073/pnas.2305625120 PMC10450430 37579155

[113] P. Mohanty , A. Rizuan , Y. C. Kim , N. L. Fawzi , J. Mittal , Protein Sci. 2024, 33, 1. 10.1002/pro.4891 PMC10804676 38160320

[114] D. Chakraborty , J. E. Straub , D. Thirumalai , Sci. Adv. 2023, 9, 10.1126/sciadv.add6921 PMC10032606 36947617

