# Supplementary material for: Transient Interdomain Interactions Modulate the Monomeric Structural Ensemble and Self‐Assembly of Huntingtin Exon 1
Source: Adv Sci (Weinh). 2025 Apr 28;12(27):2501462. doi: 10.1002/advs.202501462 (PMC12279223; doi:10.1002/advs.202501462)
Supplement: Supplementary file 1 — Supporting Information [file ADVS-12-2501462-s001.docx]

**Supporting Information**

**Transient interdomain interactions modulate the monomeric structural ensemble and self-assembly of Huntingtin Exon 1**

Priyesh Mohanty^1*^, Tien Minh Phan^1^ and Jeetain Mittal^1,2,3*^

^1^Artie McFerrin Department of Chemical Engineering, Texas A&M University, College Station, TX 77843, USA

^2^Department of Chemistry, Texas A&M University, College Station, TX 77843, USA

^3^Interdisciplinary Graduate Program in Genetics and Genomics, Texas A&M University, College Station, TX 77843, USA

*Correspondence: Priyesh Mohanty - priyeshm@tamu.edu, Jeetain Mittal - jeetain@tamu.edu

| **N17-polyQ construct** | **Box length (nm)** | **No. of replicas** | **Aggregate duration (μs)** |
| --- | --- | --- | --- |
| N17-Q_16_ - ^14^LKSF^17^ - P_5_ | 8.0 | 3 | 61.9 |
| Ac - Q_16_ - P_5_ | 8.0 | 3 | 62.1 |
| Ac - Q_16_ - NMe | 8.0 | 3 | 62.1 |
| N17 - Q_16_ - ^14^LKAA^17^ - P_5_ | 8.0 | 3 | 41.1 |
| N17 - Q_16_ - ^14^LLLF^17^ - P_5_ | 8.0 | 3 | 64.2 |
| N17 - Q_16_ - ^14^LKGG^17^ - P_5_ | 8.0 | 3 | 64.7 |
| N17 - Q_24_ - P_5_ | 8.5 | 3 | 55.4 |
| N17 - Q_32_ - P_5_ | 9.0 | 3 | 63.3 |
| N17 - Q_46_ - P_5_ (unbiased) | 10.0 | 6 | 107.2 |
| N17 - Q_46_ - P_5_ (PT-WTE) | 10.0 | 16 | 12.0  (0.75 per replica) |
| N17 - Q_16_ - PRD (PT-WTE) | 11.5 | 16 | 4.8  (0.30 per replica) |
| Ac - Q_46_ - NMe | 8.0 | 3 | 46.4 |
| N17_dimer | 8.0 | 6 | 13.7 |
| N17 - Q_7__dimer | 8.0 | 6 | 13.8 |
| N17 - Q_16_ -P_5__dimer | 8.0 | 6 | 13.5 |
| N17 - Q_16_ - P_5_ _dimer - ^14^LKGG^17^ | 8.0 | 6 | 13.9 |

**Table S1. Number of replicas and aggregate duration for N17-Q_n_-P_5_/PRD monomers and dimer trajectories generated in this study using AMBER03ws force field.**

| **Protein**  **construct** | **β-sheet**  **population (%)** |
| --- | --- |
| N17-Q_24_-P_5_ | 0.48 |
| N17-Q_32_-P_5_ | 1.82 |
| N17-Q_46_-P_5_ | 1.89 |
| Q_46_ | 0.04 |

**Table S2.** **β-sheet population computed from the aggregate trajectories of unbiased simulations for each protein construct.** For estimation of the β-sheet populations, only trajectory frames where >4 residues adopted a β-sheet conformation were considered.


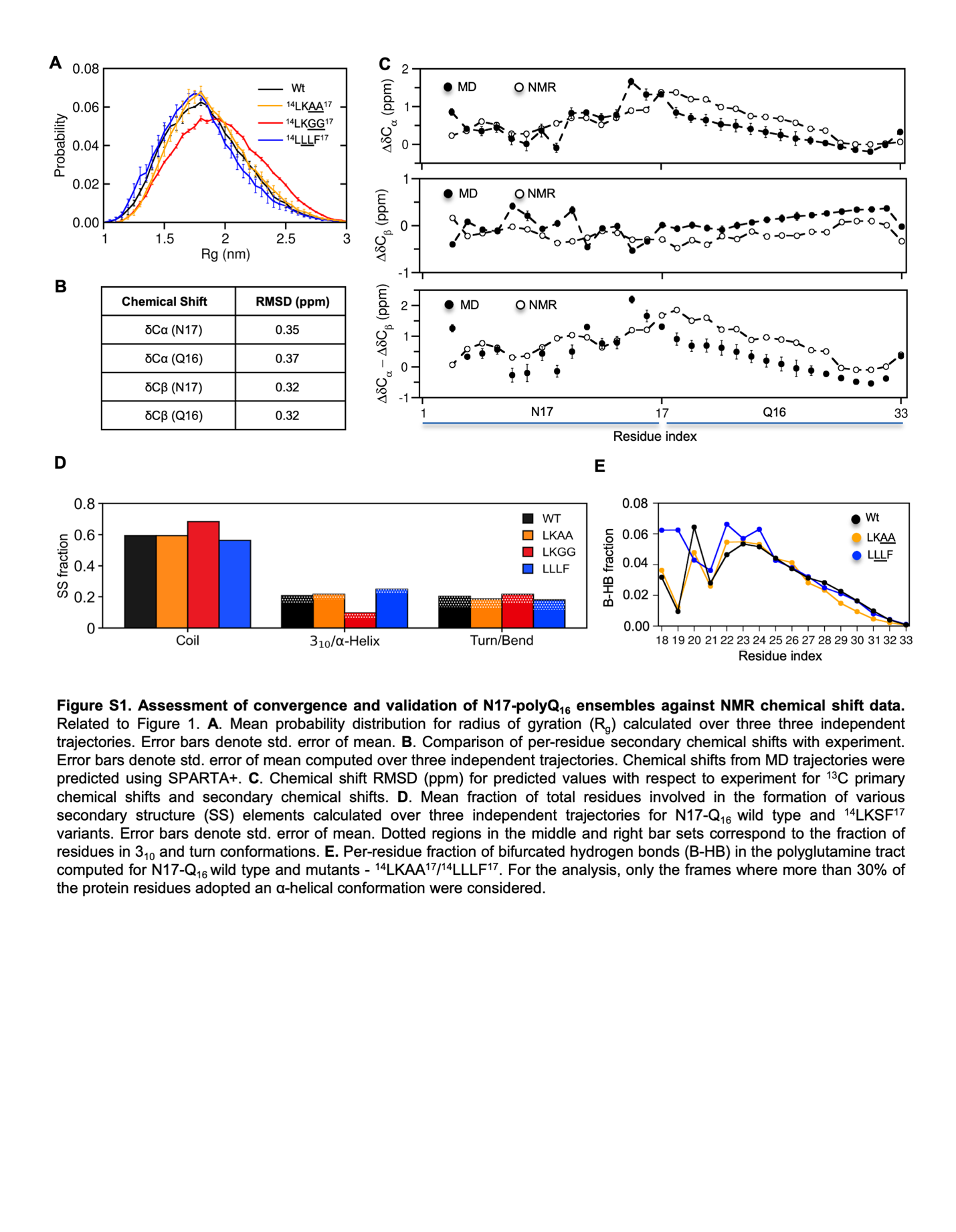


**Figure S1. Validation of N17-Q_16_-P_5_ wild-type ensemble against NMR chemical shift data and structural analysis of wild-type/mutant ensembles.** Related to Figure 1. **A**. Mean probability distribution for radius of gyration (R_g_) calculated over three independent trajectories. Error bars denote std. error of mean. **B**. Chemical shift RMSD (ppm) for predicted values with respect to experiment for ^13^C primary chemical shifts and secondary chemical shifts. Chemical shifts from MD trajectories were predicted using SPARTA+. **C**. Comparison of per-residue secondary chemical shifts with experiment. Error bars denote std. error of mean computed over three independent trajectories. **D**. Mean fraction of total residues involved in the formation of various secondary structure (SS) elements calculated over three independent trajectories for N17-Q_16_ wild type and ^14^LKSF^17^ variants. Error bars denote std. error of mean. Dotted regions in the middle and right bar sets correspond to the fraction of residues in 3_10_ and turn conformations. **E**. Per-residue fraction of bifurcated hydrogen bonds (B-HB) in the polyglutamine tract computed for N17-Q_16_ wild type and mutants - ^14^LKAA^17^/^14^LLLF^17^. For the analysis, only the frames where more than 30% of the protein residues adopted an α-helical conformation were considered.


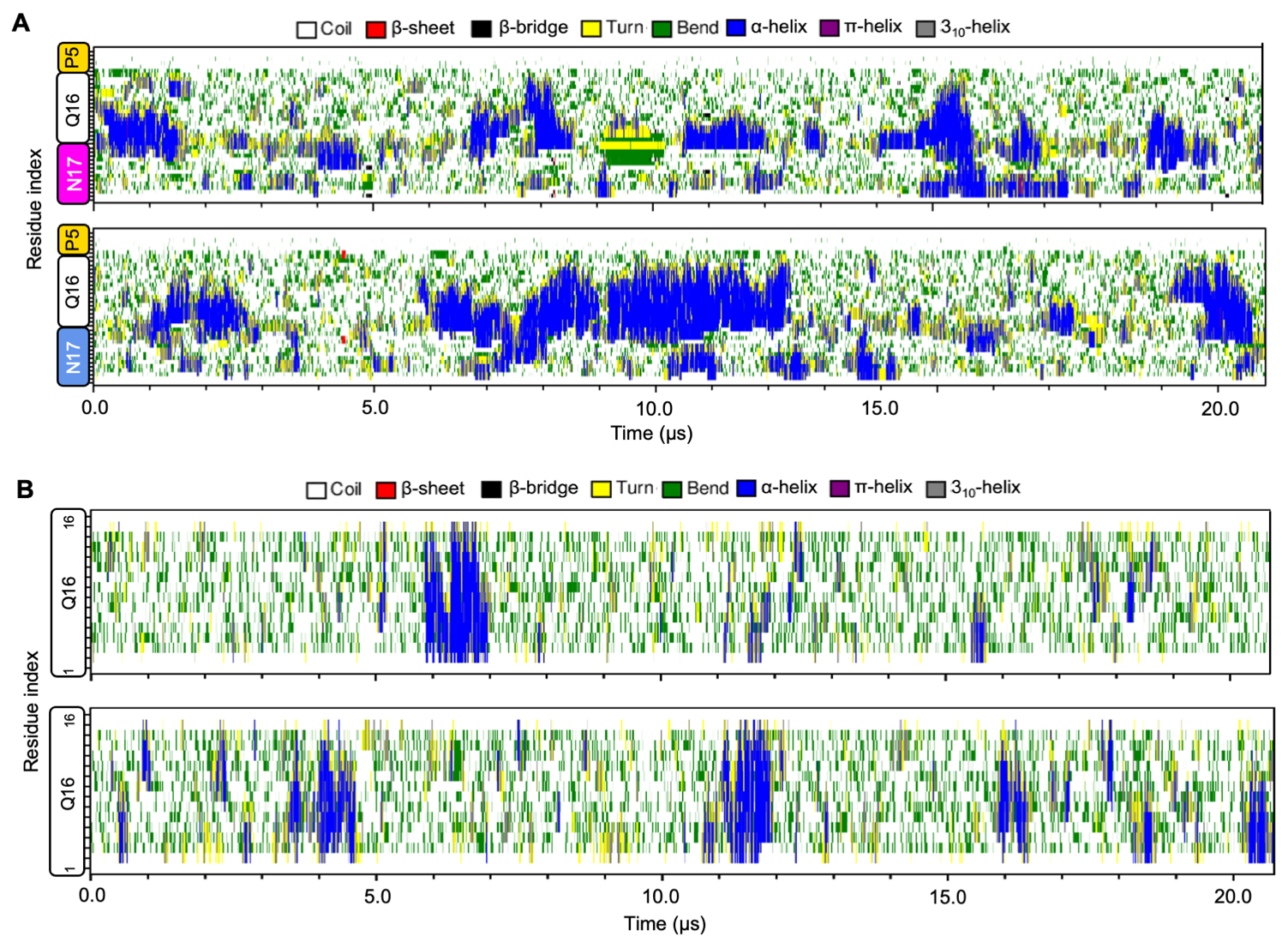


**Figure S2. Dynamics of secondary structure formation in N17-Q_16_-P_5_ and Q_16_.** Related to Figure 2. **A**. Secondary structure variation as a function of time for N17-Q_16_-P_5_ fragment from two independent trajectories. **B**. Same as in A for Q_16_.


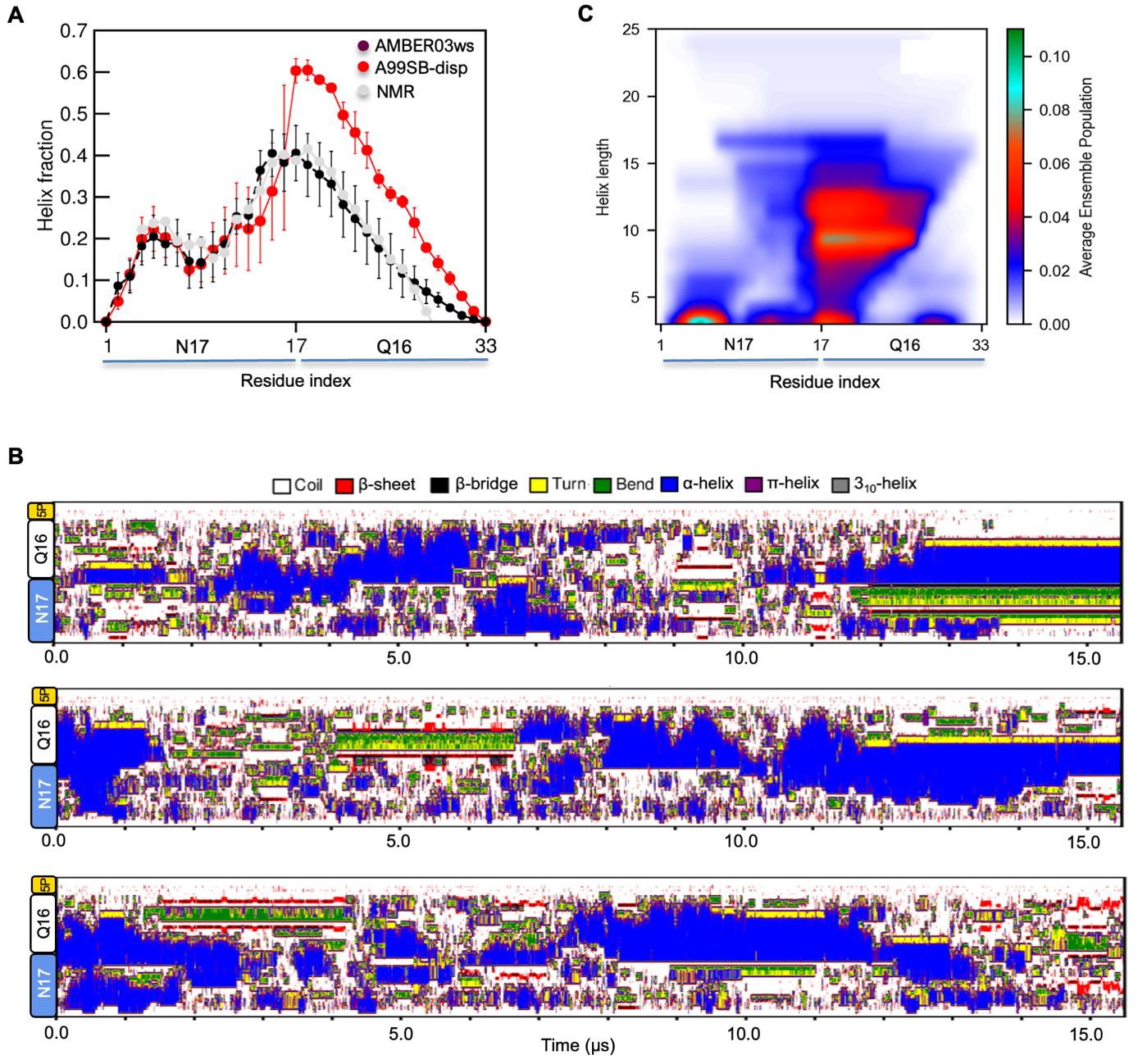


**Figure S3. Structural analysis of N17-Q_16_-P_5_ ensemble generated using AMBER99SB-disp force field.** Related to Figure 2. **A**. Comparison of mean α-helical fractions calculated over three independent AMBER99SB-disp/AMBER03ws trajectories initiated from coil conformations and their comparison to NMR SSP scores. Error bars represent SEM over three independent replicate trajectories. **B**. Secondary structure (DSSP) variation as a function of time for each trajectory. **C**. SS-map of N17-Q_16_-P_5_ wild-type computed from an aggregate trajectory (~45 μs) indicating the probability of various helical lengths across N17 and polyQ regions.

**
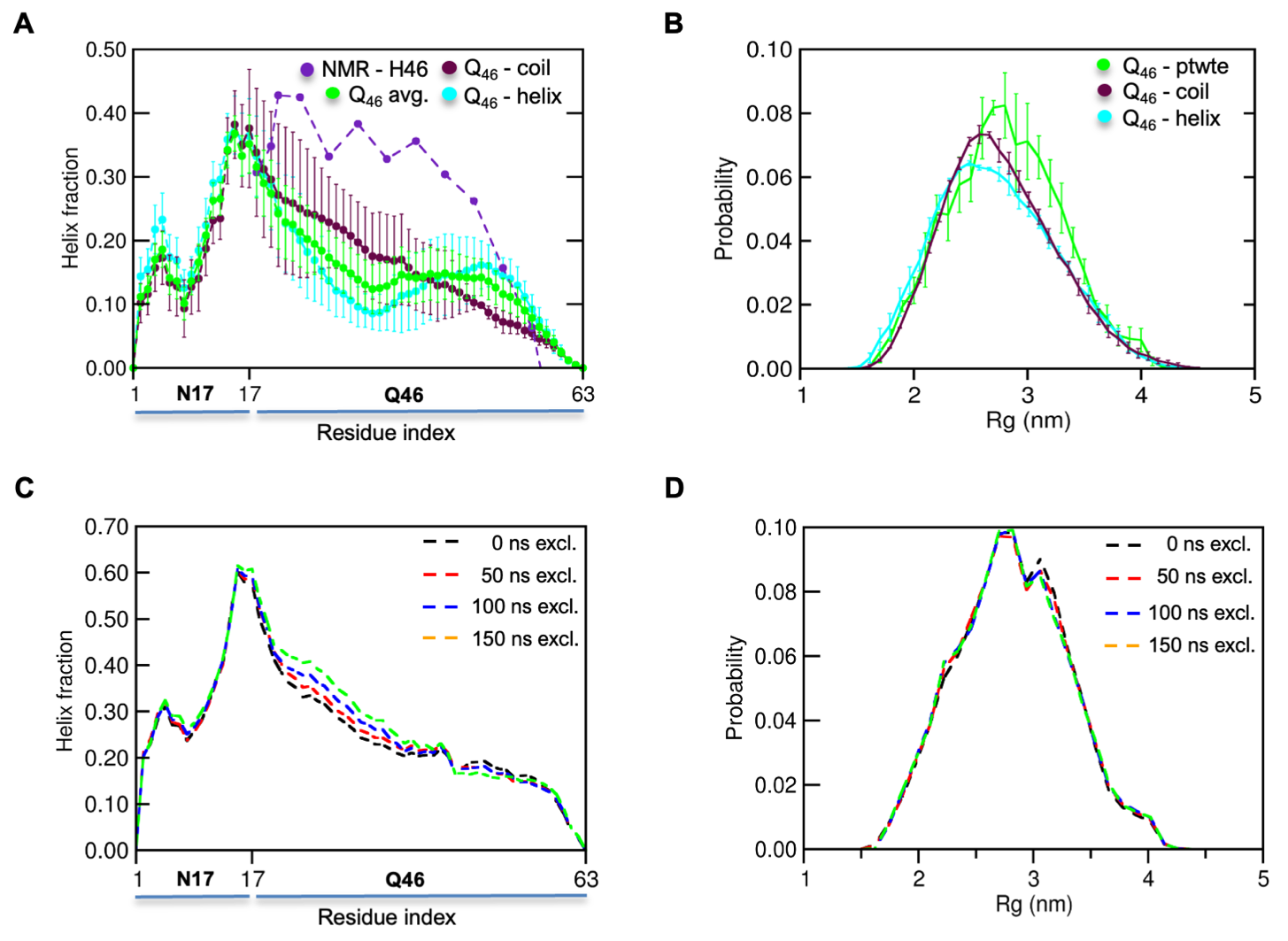
**

**Figure S4. Structural comparison of N17-Q_46_-P_5_ ensembles generated from unbiased and PT-WTE simulations.** Related to Figure 3. **A**. Mean fraction of calculated over three independent trajectories initiated from coil and partially-helical conformations. Error bars denote std. error of mean. **B**. Mean probability distributions for radius of gyration (R_g_) calculated over the unbiased trajectories and the PT-WTE trajectory (500 ns) at 293.15 K. Error bars denote std. error of mean calculated over independent trajectories for coil/helix and 4 blocks (150 ns) of the PT-WTE trajectory. **C.** Per-residue ɑ-helical fractions **D.** radius of gyration (R_g_) probability (right) distributions (dashed lines) of 293 K replica ensembles calculated over the whole 750 ns trajectory (black), first 50 ns (red), first 100 ns (blue) and first 150 ns (green) excluded overlap indicating good convergence of local structure and global dimensions respectively.

**
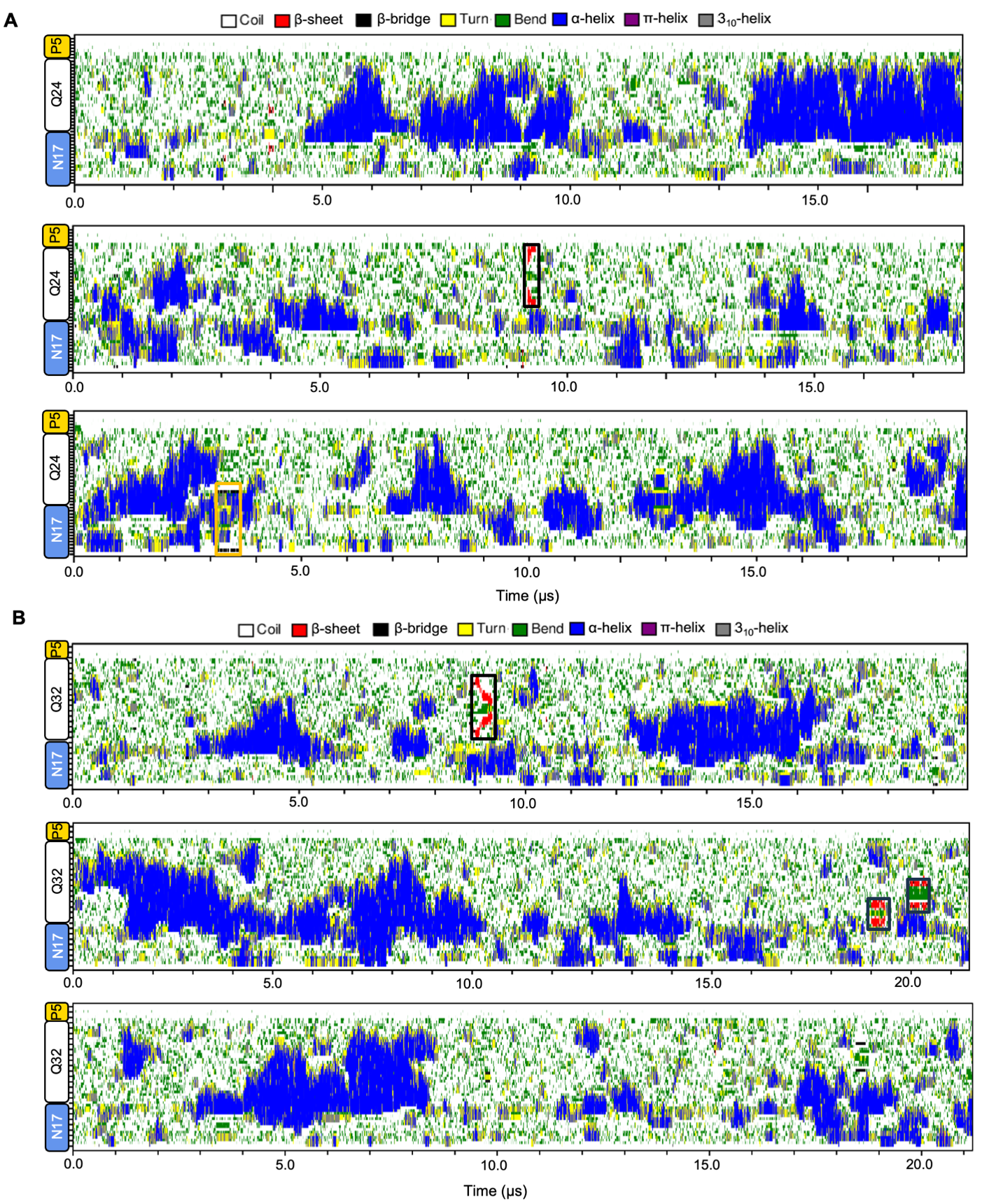
**

**Figure S5. Secondary structure dynamics in N17-Q_24/32_-P_5_ trajectories.** Related to Figure 3.

**A.** DSSP secondary structure variation as a function of time for three N17-Q_24_-P_5_ trajectories initiated from random coil conformation. **B.** Same as in A for N17-Q_32_-P_5_. In both A and B, β-bridge/sheet structures are highlighted in black boxes.


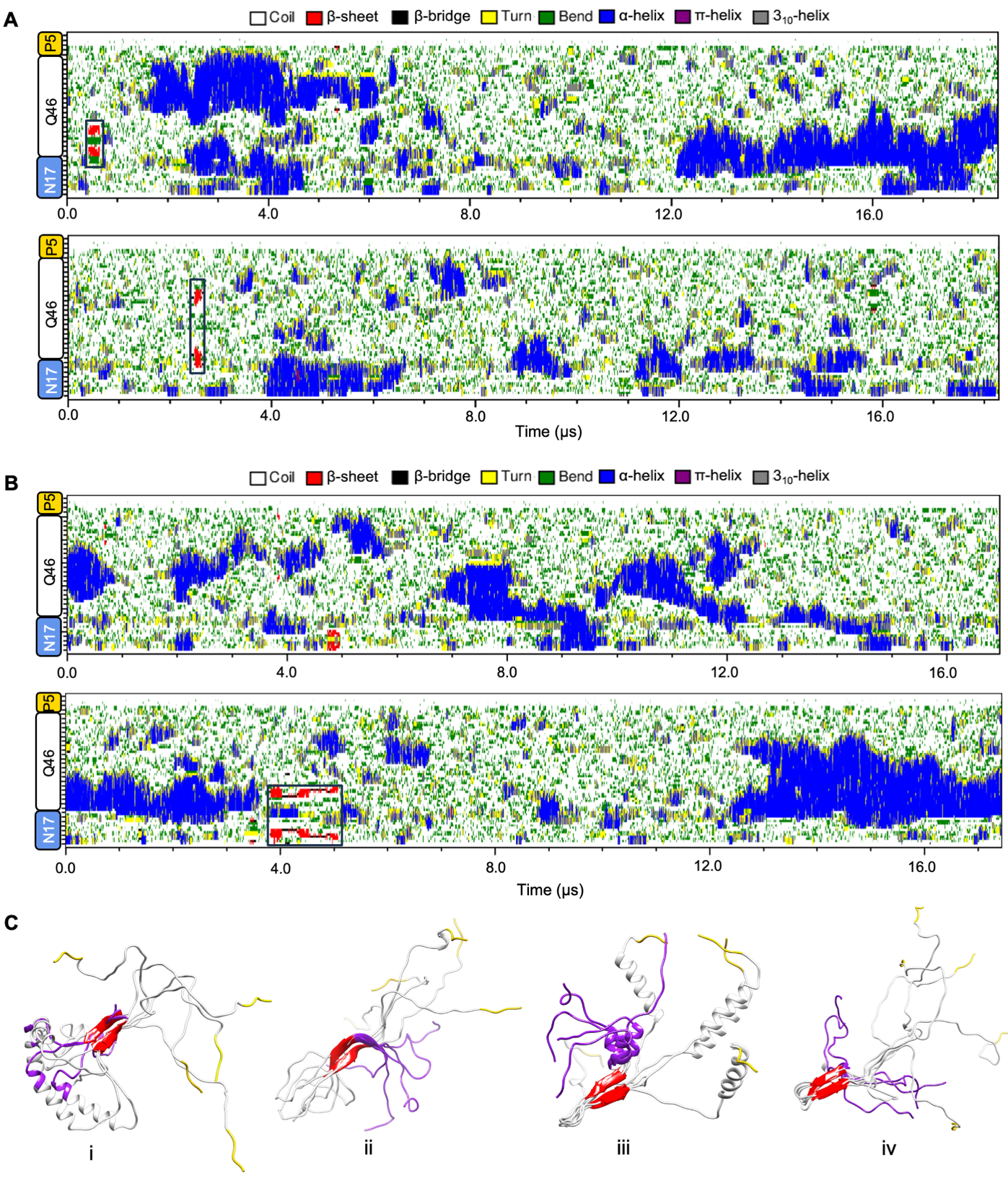


**Figure S6. Secondary structure dynamics in N17-Q_46_-P_5_ trajectories.** Related to Figure 3.

**A.** Secondary structure variation as a function of time for unbiased N17-Q_46_-P_5_ trajectories initiated from random coil conformation. β-sheet structures formed are highlighted in black boxes. **B**. Same as in A for two trajectories initiated from partially helical conformations. **C**. β-sheet ensembles (i - iv) from trajectory intervals highlighted in black boxes in panels A/B and Figure 2C. Structures i/ii formed N17/polyQ β-sheets while iii/iv formed intra-polyQ β-sheets. Five representative structures are shown for each ensemble. The coloring scheme for the structures is as follows: N17 - purple, Q46 - white and 5P - Gold, β-strands - red.

**
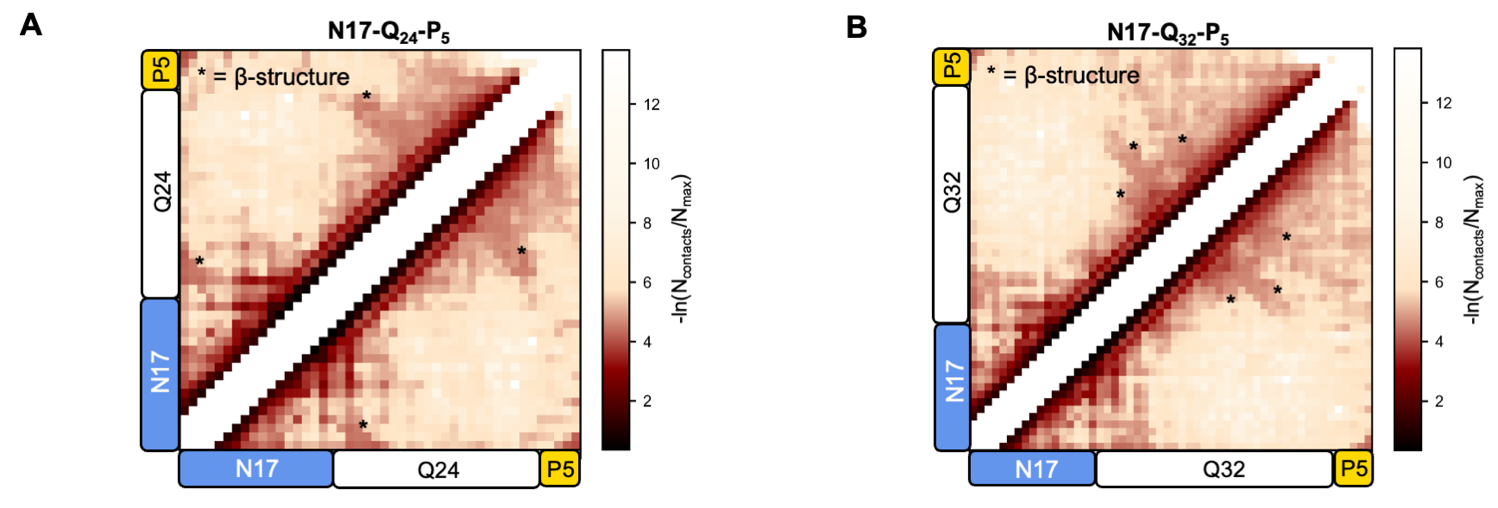
**

**Figure S7. Contact analysis of N17-Q_24/32_-P_5_ ensembles indicates transient β-sheet conformations.** Related to Figure 3. **A**. Two-dimensional intramolecular contact maps calculated over three independent N17-Q_24_-P_5_ trajectories highlighting the transient (low) population of β-sheet conformations (marked as *) relative to α-helices in the ensemble. **B.** Same as in A for N17-Q_32_-P_5_ trajectories.

**
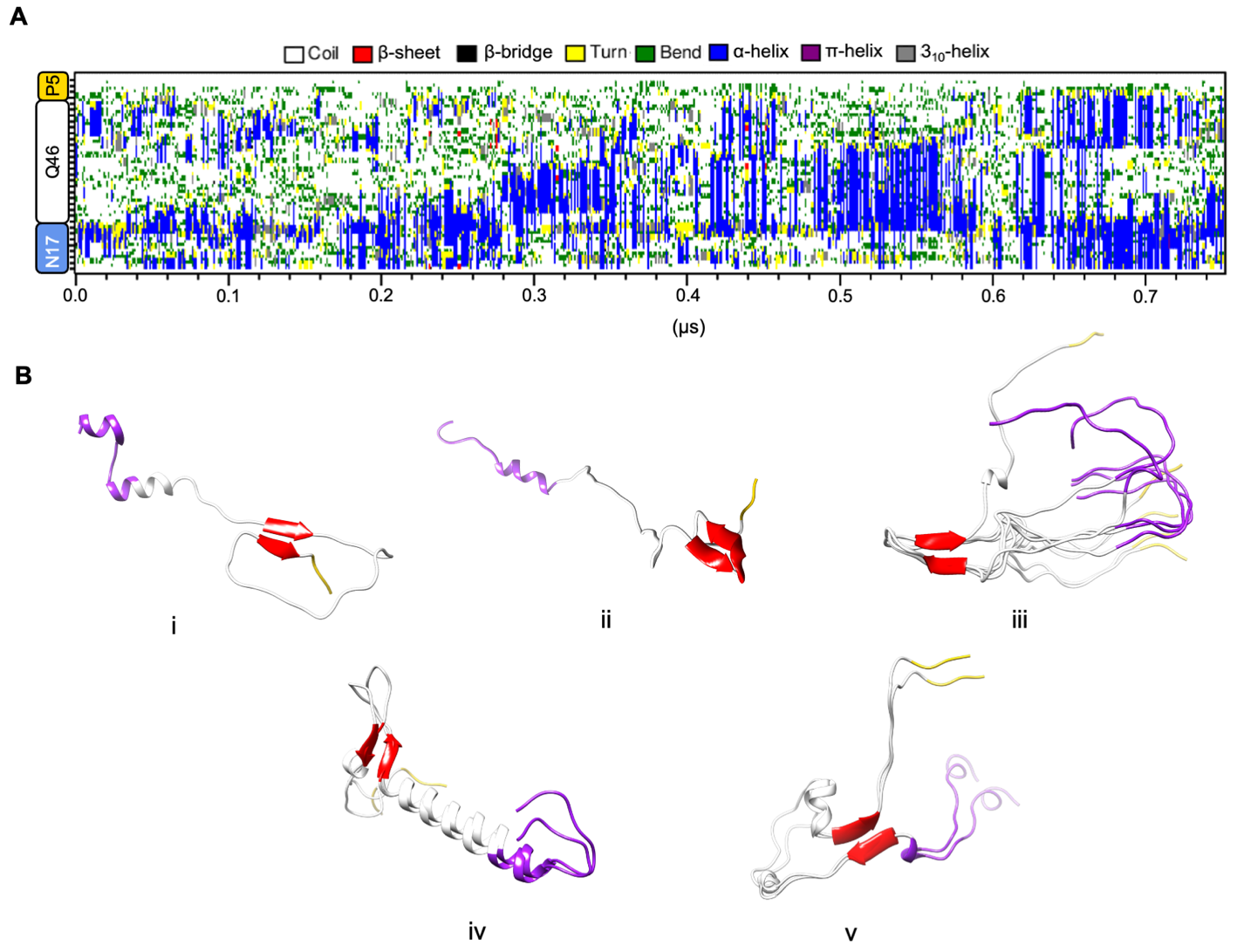
**

**Figure S8. Secondary structure analysis of the 293 K replica trajectory for N17-Q_46_-P_5_ from PT-WTE simulations.** Related to Figure 3. **A.** Secondary structure variation as a function of time for N17-Q_46_-P_5_ from replica trajectory. **B**. Representative β-sheet structures of clusters (i-vi) which transiently form in the replica trajectory over 600 ns (first 150 ns excluded as equilibration time) analyzed every 100 ps. The coloring scheme is as described for Fig. S6C.

**
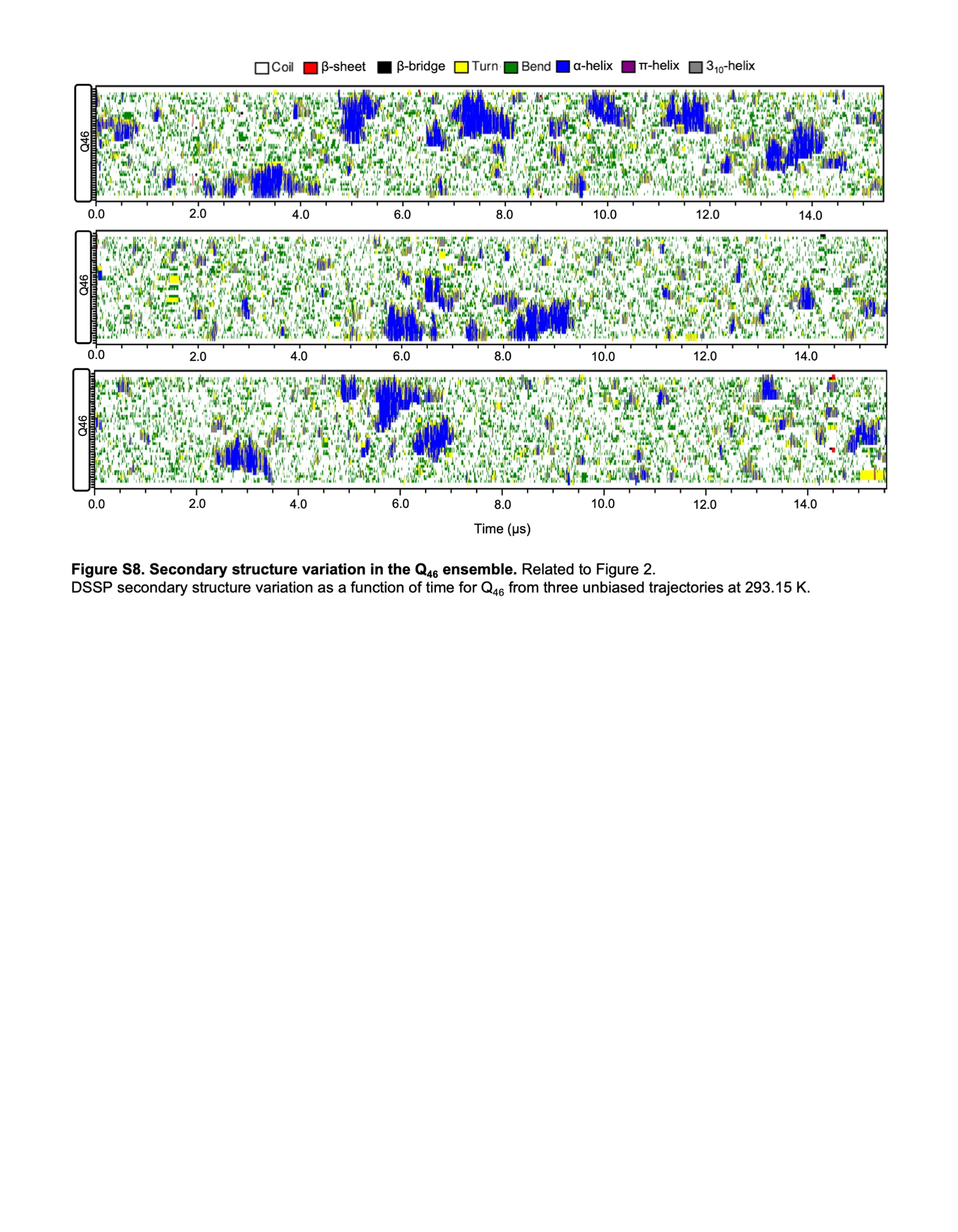
**

**Figure S9. Secondary structure variation in the Q_46_ ensemble.** Related to Figure 3.

DSSP secondary structure variation as a function of time for Q_46_ from three unbiased trajectories at 293.15 K.

**
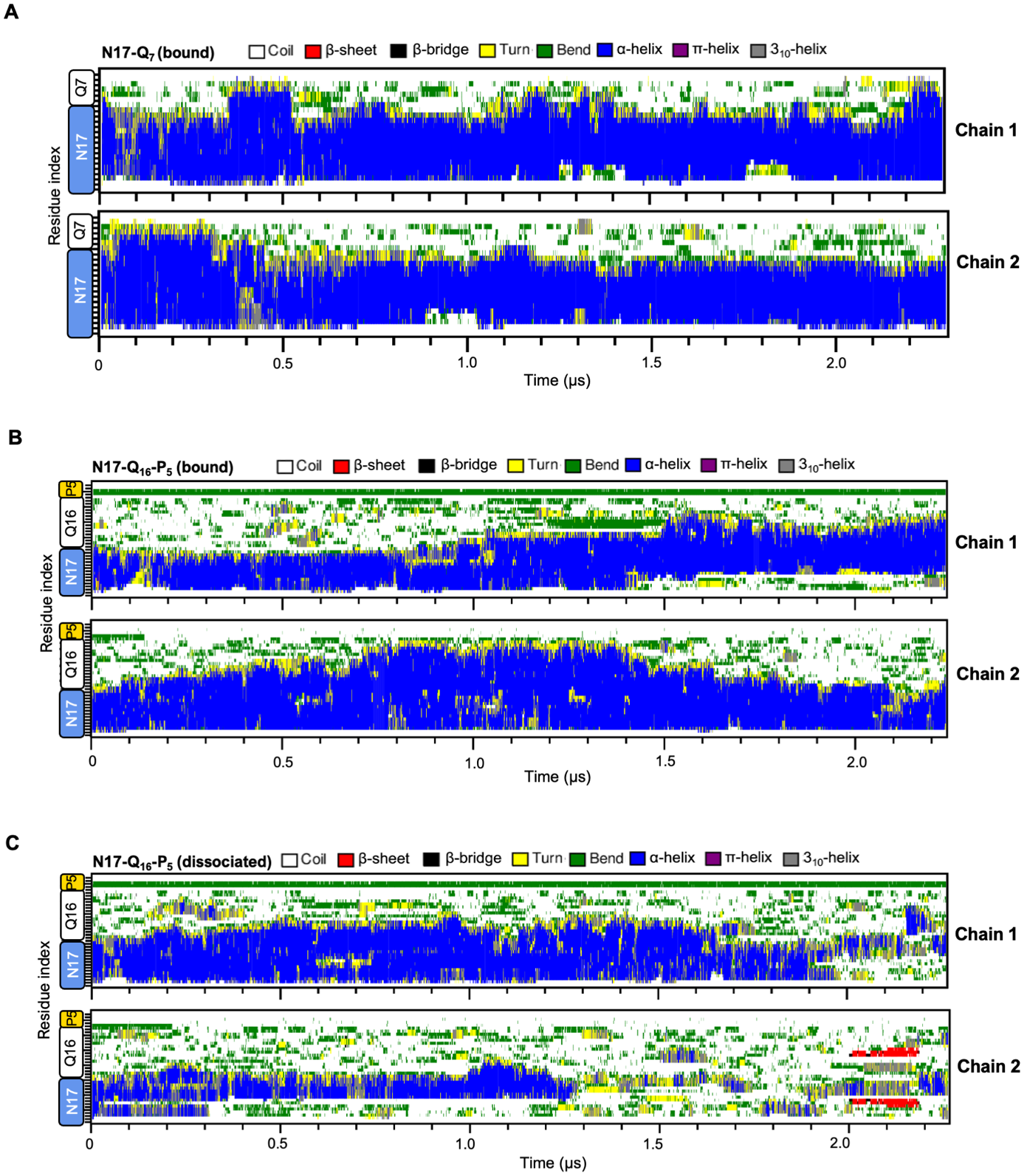
**

**Figure S10. Effect of dimerization on N17-Q_7/Q16_ secondary structure stability.** Related to Figure 4**A.** Secondary structure variation as a function of time for each chain in a representative N17-Q_7_ dimer trajectory which shows stable association. The plot highlights the stabilization of α-helical conformations in the bound dimer. **B.** Same as in A for C for N17-Q_16_-P_5_. **C.** Secondary structure variation as a function of time for each chain in a representative N17-Q_16_-P_5_ dimer trajectory which undergoes dissociation after 1.3 μs. The plot highlights the destabilization of α-helical conformations upon dimer dissociation.


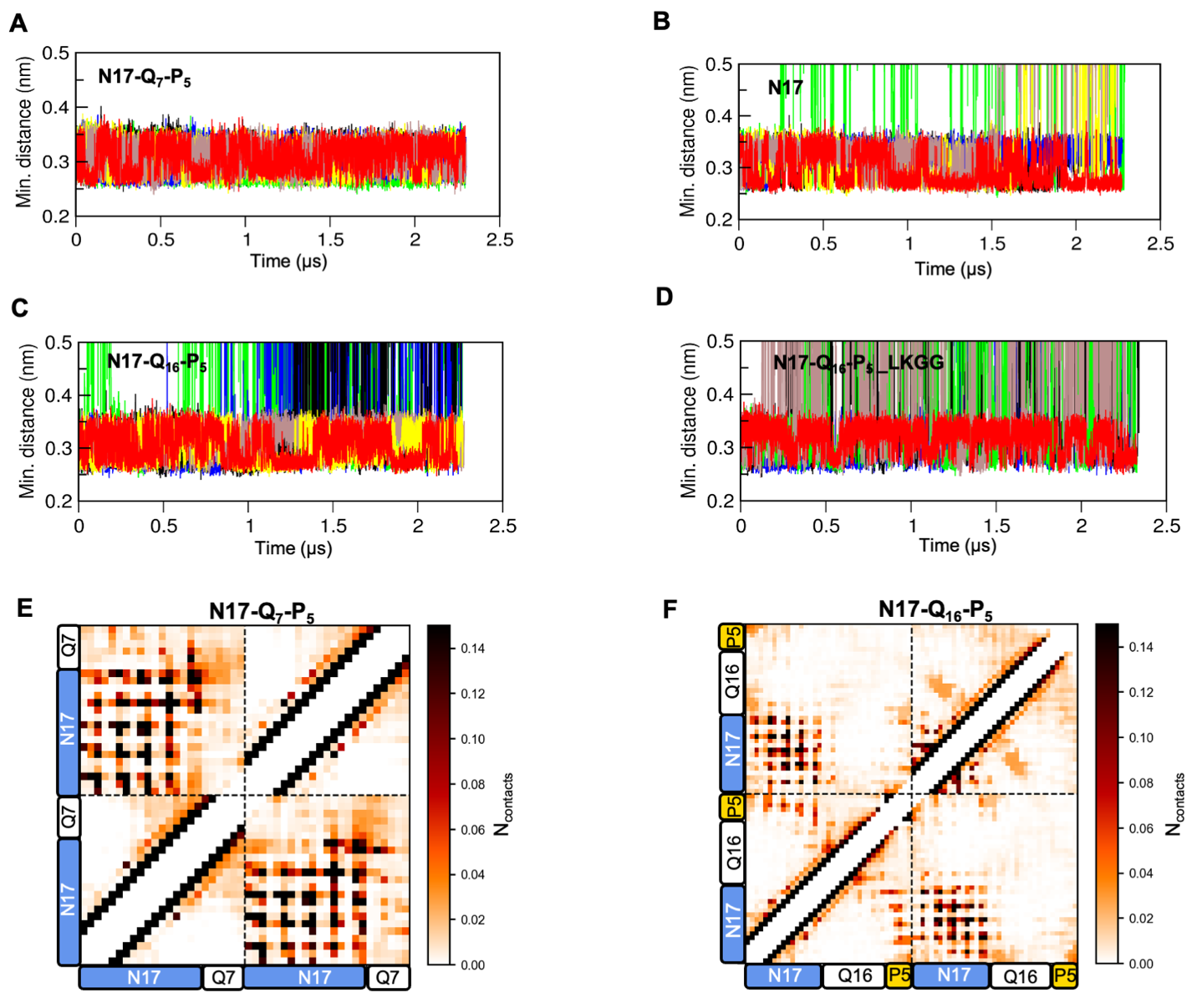


**Figure S11. Minimum distance and pairwise contact analysis to assess the effect of domain “cross-talk” on dimer stability.** Related to Figure 4. **A.** Minimum distance analysis as a function of time between monomer units of N17-Q_7_ dimer. **B.** Same as in A for N17 dimer. Time points where the minimum distance first exceeds 0.4 nm corresponds to complete dissociation of the dimer. **C.** Same as in B for N17-Q_16_-P_5_. **D .** Same as in B for N17-Q_16_-P_5__LKGG variant. **E**. 2D contact map highlight the network of weak inter-domain interactions within the N17-Q_7_ dimer. The contact map was averaged over six independent trajectories. **F**. Same as in panel B for the N17-Q_16_-P_5_ dimer.


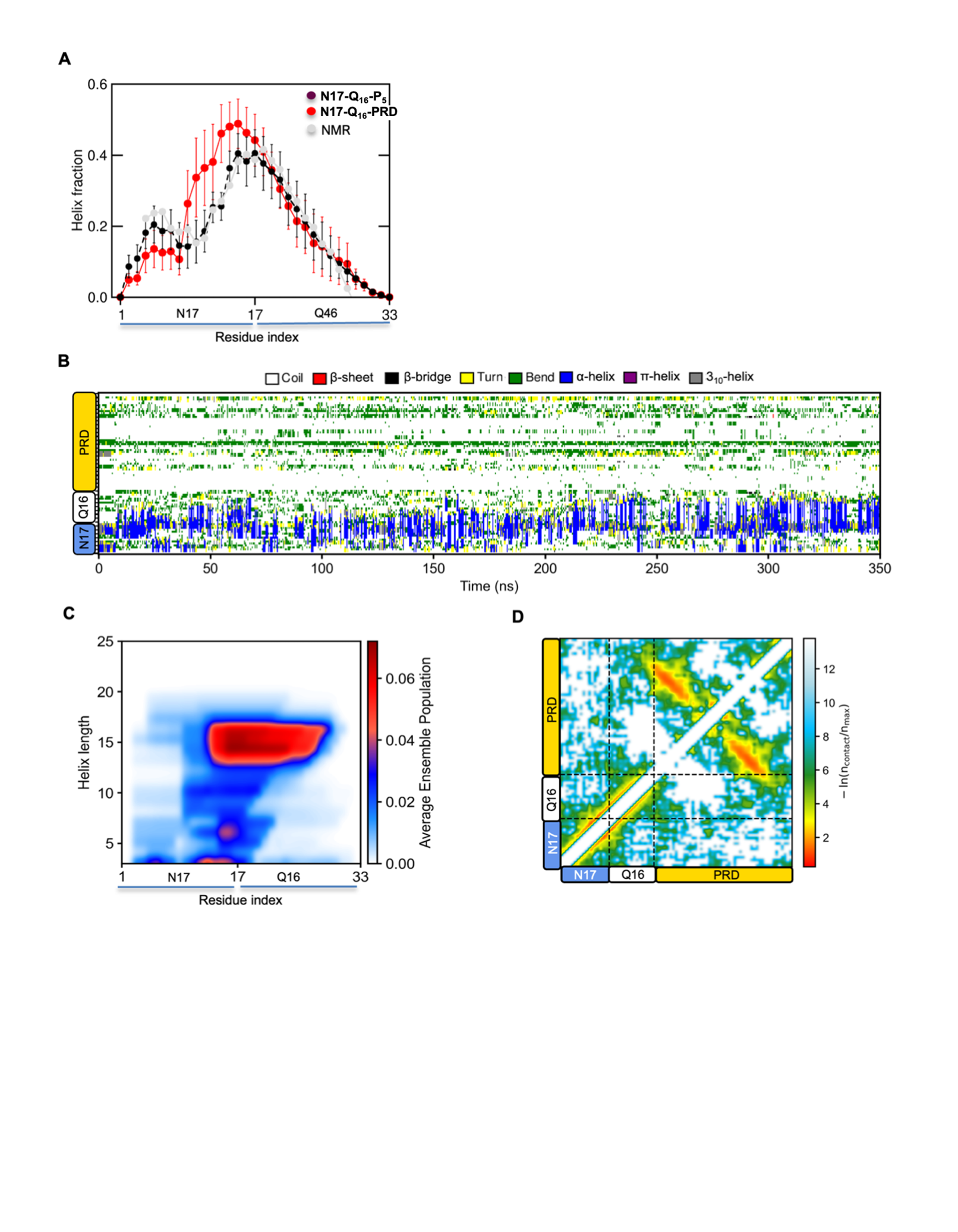


**Figure S12. Structure ensemble and intradomain interactions of N17-Q_16_-PRD computed from PT-WTE simulations.** Related to Figure 5. **A**. Per-residue ɑ-helical fraction of Httex1 (293 K replica) and its comparison with N17-Q_16_ and NMR SSP scores. The first 100 ns was excluded as equilibration time. The error bars for Httex1 correspond to SEM over 4 independent blocks (62.5 ns each) of the 200 ns trajectory. **B**. DSSP secondary structure variation as a function of time for the 293 K replica trajectory. **C**. SS-map of Httex1-Q_16_ (left) computed from the 293 K replica trajectory (excluding first 50 ns) indicating the probability of various ɑ-helical lengths across N17 and polyQ regions. **D**. 2D-intramolecular contact map analysis of the Httex1 (293 K) ensemble highlighting the prevalence of PRD intradomain interactions involving two polyproline tracts (P_10_/P_11_).


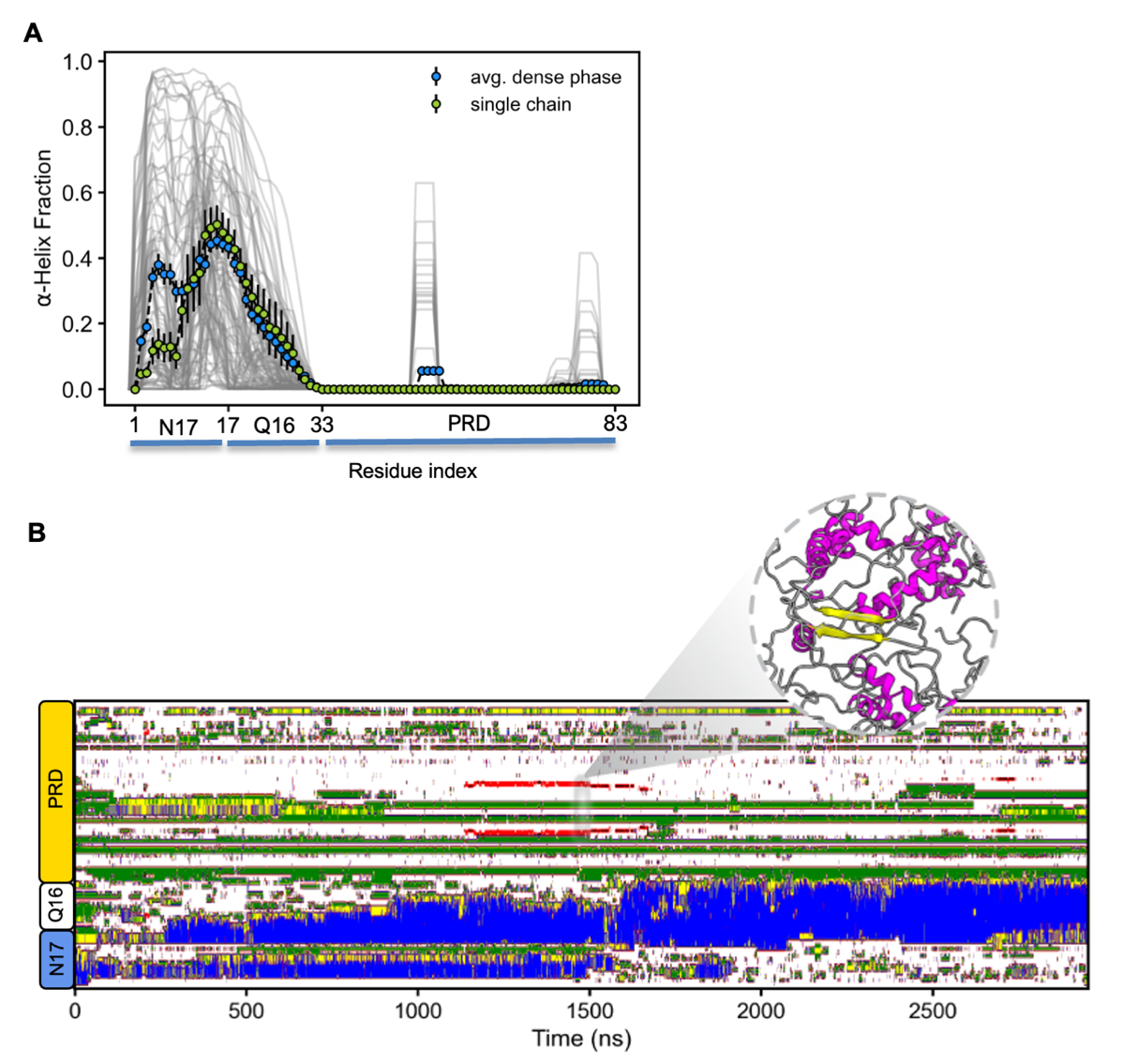


**Figure S13. Structural analysis of N17-Q_16_-PRD condensate.** Related to Figure 5.

**A**. Mean ɑ-helical fraction per residue over all condensed phase chains reveals a substantial enhancement in N17 ɑ-helicity (aa: 2-11) compared to single chain (monomer). **B**. DSSP secondary structure variation as a function of time for a representative molecule within the condensate which forms a transient β-sheet from 1.2 to 1.5 us.
